# LecB from *Pseudomonas aeruginosa* modulates Piezo1 currents and localization in a time-dependent manner

**DOI:** 10.1007/s00018-025-05934-z

**Published:** 2025-11-14

**Authors:** Anna-Sophia Kittel, Olga Makshakova, Michael Hauerwas, Nikita Edel, Niklas Knickmeier, Jana Tomisch, Ahmad Aljohmani, Daniela Yildiz, Rémi Peyronnet, Winfried Römer

**Affiliations:** 1 Faculty of Biology, University of Freiburg, Freiburg, Germany; 2 Signalling Research Centres BIOSS and CIBSS, University of Freiburg, Schänzlestraße 18, Freiburg, 79104 Germany; 3 Faculty of Chemistry and Pharmacy, University of Freiburg, Freiburg, Germany; 4 Institute of Experimental and Clinical Pharmacology, PZMS, ZHMB, Saarland University, Homburg, Germany; 5 Faculty of Medicine, University of Freiburg, Freiburg, Germany; 6 Institute for Experimental Cardiovascular Medicine, Elsässer Str. 4C, Freiburg, 79110 Germany

**Keywords:** Lectin, Ion channel, Protein-carbohydrate interaction, Glycosylation, Cell migration, Bacterial virulence factor, Patch clamp, Computer simulation

## Abstract

Infections with the Gram-negative opportunistic pathogen *Pseudomonas aeruginosa* are becoming increasingly difficult to treat due to growing antibiotic resistance. Complications often include disturbed wound healing and impaired cell migration of various cell types, including epithelial and immune cells in the host tissue. One bacterial virulence factor responsible for these effects is the carbohydrate-binding lectin LecB. It mediates adhesion to host cells, alters various cellular signaling pathways and internalizes several receptors, i.e. integrins. However, the full effects and mechanisms of how LecB influences the processes in the host cells are still largely unknown. In this study, we introduce a new host cell interaction partner of LecB with strong physiological impact. Using immunofluorescence and pull-down studies, we were able to show that LecB can interact with the cation nonselective stretch-activated channel Piezo1, which is expressed in various cell types. Recording Piezo1 currents with the patch-clamp technique in the in presence of LecB, we observed altered responses of Piezo1 to mechanical forces. After 30 min of LecB incubation time, mechanically-induced Piezo1 currents were higher compared to control, while after 3 h they were greatly reduced. Computational modeling suggests protein–protein and protein-carbohydrate interactions between LecB and Piezo1. This hypothesis is supported by inhibiting LecB-induced current changes by l-fucose or a LecB binding site mutant. From a more general perspective, our results highlight ion channels and their glycosylations as targets for bacterial lectins, improving our understanding of host–pathogen interactions and the evolution of bacterial infections, and hopefully providing the basis for the development of new therapeutics to combat antibiotic-resistant pathogens.

**Supplementary Information:**

The online version contains supplementary material available at 10.1007/s00018-025-05934-z.

## Introduction

Antibiotic-resistant bacteria pose an increasing threat to human health. According to the United Nations, 10 million deaths per year are expected worldwide by 2050 due to infections caused by such pathogens [1]. One prominent candidate is the invasive, Gram-negative bacterium *Pseudomonas aeruginosa (P. aeruginosa)*, which causes wound healing disorders and impaired cell migration in surgical wounds and burns. It can also lead to acute pneumonia in immunocompromised and cystic fibrosis patients [2–4]. For infection, it is equipped with a broad variety of virulence factors [5–7]. This includes the ability to form biofilms [8], and express flagella, pili [9–11] and exotoxins [12–15]. *P. aeruginosa* has a quorum sensing system [16–18] and the ability to develop antibiotic resistances through efflux pumps, porins or β-lactamases [6, 19–23], which makes it difficult to cure such infections. In addition, *P. aeruginosa* expresses the two carbohydrate-binding lectins LecA (also named as PA-IL) and LecB (also named as PA-IIL) [25]. Both proteins are homo-tetramers with one carbohydrate binding pocket per monomer and have no proteolytic activity [25–27]. LecA mainly binds to D-galactose, and in particular to the host cell glycosphingolipid globotriaosylceramide (also known as Gb3), which is overexpressed in some types of cancer [24, 29]. Furthermore, LecA induces negative membrane curvature and enables bacterial invasion via a lipid zipper mechanism [28, 29]. LecB has a broader spectrum of ligands, in particular l-fucose, and to a lesser extent, d-mannose patterns, which are widely used in glycosylation of proteins and lipids at the plasma membrane [30]. It has been published that LecB targets several important molecules at the host cell plasma membrane, e.g. β1-integrins and insulin-like growth factor receptor 1 (IGF1R), and leads to their internalization and transport along the degradation pathway, resulting in e.g. loss of cell polarity and tissue integrity in polarized epithelial cells or loss of cell fitness in keratinocytes [32–34]. LecB has also been reported to alter host cell signaling pathways and impair cell migration in epithelial cells in a concentration-dependent manner [33]. This effect was reversible by either washing out or neutralizing LecB with l-fucose or tailored carbohydrate-based inhibitors [32–35, 80]. Recent findings emphasize the importance of LecB as a bacterial virulence factor that suppresses immune responses by blocking dendritic cell migration and T cell proliferation in in vivo models [36].

Since LecB influences a variety of processes in host cells, it contributes significantly to the pathogenicity of *P. aeruginosa*. Unfortunately, the molecular mechanisms of these processes, including the key molecules, are still completely unclear. In this study, we wanted to identify further important interaction partners of LecB in epithelial cells that are associated with cell migration. A mass spectrometry analysis carried out in human keratinocytes formed the basis for further investigations. LecB-biotin-treated primary keratinocytes were mildly lysed to conserve LecB-induced plasma membrane domains and subsequently treated with streptavidin-beads and analyzed via mass spectrometry, as described previously [34]. The results showed the cation non-selective channel Piezo1 as a promising candidate for interactions with LecB. Piezo1 is a propeller-shaped homo-trimer and belongs to the stretch-activated ion channel (SAC) family as membrane tension directly affects its gating [37–39]. Upon activation, Ca^2+^ ions and other cations are non-selectively conducted through the channel pore into the cell. Piezo1 is expressed in many mammalian cell types and plays crucial physiological roles in diverse tissues, including mechano-sensation of shear flow in blood vessels [40], modulation of calcium signaling in cardiomyocytes [41, 42], and integrin maturation and cytoskeleton arrangement [83]. Piezo1 has been reported to associate with integrins and other cellular adhesion molecules (CAMs), such as platelet endothelial cellular adhesion molecule-1 (PECAM-1), particularly in adherens junctions, thereby strengthening tissue integrity and enabling stretch-induced remodeling of adherens junctions in epithelial cell monolayers [43, 82]. Furthermore, Piezo1 has been shown to regulate cell migration of keratinocytes during the wound healing process [44] and to act as an immune sensor in a mouse lung infection model. Its expression is crucial for innate immune signaling [45] and its dysregulation is also associated with various diseases, such as cardiac hypertrophy [46], cancer [47, 81] and fibrosis [48].

In this study, we describe the impact of the *P. aeruginosa* lectin LecB on SAC activity and in particular on Piezo1 upon activation by stretch using the patch-clamp technique in the cell-attached configuration of wild-type (WT) epithelial cells (H1299 and HaCaT cells) and EGFP-mPiezo1-overexpressing H1299 cells. We detected higher mechanically induced Piezo1 currents after 30 min of LecB incubation and a largely reduced current at a later time point (i.e. 3 h) after addition. Using immunofluorescence, we showed that LecB has an effect on the distribution of Piezo1 and partially co-localizes with Piezo1, especially at cell–cell contacts for early time points and at the rear of the cells for later time points. These effects were not occurring when LecB was incubated together with l-fucose or when a LecB mutant with impaired binding properties to l-fucose, was used instead of wild-type LecB [49]. Furthermore, using pull-down assays, we were able to show that the binding of LecB to carbohydrates is essential for the interaction of LecB with Piezo1. Computer modeling suggests that these interactions may occur in the cap regions of Piezo1 in close proximity to N-glycosylation sites. These data suggest that Piezo1 is a putative target of LecB, potentially leading to an impairment of cellular processes such as cell migration, wound healing and inflammatory signaling that allows *P. aeruginosa* to establish epithelial infection.

## Materials and methods

### Cell culture, EGFP-mPiezo1 overexpression and knockdown

H1299 non-small lung cancer epithelial-like cells (American Type Culture Collection, CRL-5803) were grown in Roswell Park Memorial Institute (RPMI) 1640 medium supplemented with 2 mM L-glutamine (Gibco, ThermoFisher Scientific, Waltham, MA, USA) and 10% fetal calf serum (Gibco, ThermoFisher Scientific, Waltham, MA, USA) and incubated at 37 °C and 5% CO_2_ until confluence was reached. For overexpression studies, 2.5 * 10^4^ cells were seeded in 35 mm diameter dishes. Per dish, 0.5 µg DNA was diluted in 50 µL 150 mM NaCl and 1.5 µL of linear jetPEI (linear, MW 25000, transfection grade, Polysciences) was diluted in another 50 µL 150 mM NaCl. After 5 min of incubation at room temperature, the solutions were mixed and after additional incubation of 15 min, then drop-wisely added to the seeded cells. Cells were incubated at least for 48 h and maximum 72 h until electrophysiology, Western Blot or immunocytochemistry experiments were performed. For overexpression, the plasmids mPiezo1-pEGFP-N1 encoding murine Piezo1 fused to EGFP, and pIRES-EGFP encoding EGFP, kindly provided by Eric Honoré, were used [50, 51].

To decrease the level of endogenous Piezo1 in H1299 cells, siRNA-mediated knockdown was performed. 2 * 10^4^ cells were seeded in 35 mm diameter dishes and adhesion to the culture vessel was allowed by incubation at 37 °C overnight. The respective siRNA (siPiezo1 and non-targeting (siNT) as a control) were diluted in serum-free RPMI1640 medium to a final concentration of 8 nM, and 5 µL of HiPerfect were added per transfected culture dish. After 15 min of incubation at RT, the transfection mix was added to the cells. After 72 h, patch-clamp and Western Blot experiments were performed. The siRNA directed against Piezo1 (Entrez Gene 9780) and non-targeting (NT) were purchased from Horizon Discovery (SMART-pool ON-TARGET plus). Human immortalized keratinocytes (HaCaT) cells (Cytion, #300493) were grown in Dulbecco’s modified Eagle’s Medium (DMEM), containing 4.5 g/L d-glucose, supplemented with 2 mM L-glutamine and 10% FCS and incubated at 37 °C and 5% CO_2_ until confluence was reached. Cells were passaged and seeded as described above for H1299 cells.

### LecB production

Production of recombinant *P. aeruginosa* LecB (UniProt ID: Q9HYN5_PSEAE) and LecB-S22A mutant (PDB 00002jdm) was performed in *Escherichia coli* BL21(D3), transformed with pET25b vector. The pET25b_S22A vector was kindly provided by Anne Imberty (CNRS, Grenoble, France). Both proteins were purified by chromatography with a mannose-agarose (#M6400, Sigma Aldrich) column, as previously stated elsewhere [31, 52, 53]. Dialysis against PBS was performed for at least 4 times. The proteins were concentrated, snap-frozen and stored at −80 °C. Purity of the protein was determined via SDS PAGE and Western Blot, Ponceau staining of the membrane and antibody staining against LecB. Furthermore, the A280/A230 ratio was determined spectrophotometrically to test purity of the sample.

### Lectin labeling with a fluorophore

LecB, dissolved in 1 × PBS, was labeled with Alexa Fluor™ 647 via succinimidyl ester conjugation. The protein solution was diluted with NaHCO_3_ (1 M, pH 8.5, diluted in ddH_2_O) in a ratio of 1:10. The fluorophore was dissolved in DMSO in a concentration of 10 μg/ml and the volume of dye solution required for the amount of protein was adjusted to a molar excess of 5. The reaction was incubated for 1 h at room temperature with continuous stirring. A Zeba Desalting spin column was prepared by removing of column bottom closure and centrifuging at 1,500 × g for 1 min to remove the storage solution. The column was centrifuged twice at 1,500 × g for 1 min by adding of 300 μL 1 × PBS to wash the column. Afterwards, the column was placed in a new collection tube and 30—130 μL of sample was applied on the top of the resin bed and centrifuged again at 1,500 × g for 2 min to collect the sample. The final concentration of the labeled protein was measured with NanoDrop 2000 (ThermoFisher Scientific).

### Lectin biotinylation

Sulfo-NHS-SS-biotin (ThermoFisher Scientific) was added to a lectin solution with a molar excess of 20 and incubated for 2 h at 4 °C. Non-reacted Sulfo-NHS-SS-biotin was removed by dialysis using a Slide-A-Lyzer MINI Dialysis Cassette (ThermoFisher Scientific). The concentration of biotinylated LecB was measured with NanoDrop 2000 (ThermoFisher Scientific). A HABA assay (ThermoFisher Scientific) was performed to quantify the amount of biotin that was bound to LecB.

### Patch-clamp recordings and mechanical stimulation

As described above, H1299 cells or HaCaT cells were cultivated in 35 mm dishes. H1299 cells were transiently transfected overexpressing EGFP-mPiezo1. Cells were incubated either in pure cell culture medium or in culture medium containing 1 µM LecB for 30 min or 3 h prior the experiment. The patch-clamp technique was used to assess Piezo1 currents in the plasma membrane. Cell-attached patch-clamp recordings were performed as previously described [54]. The bath solution was composed of 155 mM KCl, 5 mM EGTA, 10 mM HEPES and 3 mM MgCl_2_ with a pH of 7.2 and 300 mosmol/L. The pipette solution contained 150 NaCl, 5 mM KCl, 10 mM HEPES and 2 mM CaCl_2_ with a pH of 7.4 and 300 mosmol/L. Patch pipettes were pulled of fire-polished soda lime glass capillaries (inner diameter 1.15 ± 0.05 mm, outer diameter 1.5 ± 0.05 mm, VITREX Medical, Denmark) to achieve a pipette resistance of 1.1–1.3 MΩ. Cells were placed on an inverted epifluorescence microscope and were approached with the pipette under positive pressure ranging from 5–10 mmHg. To achieve a seal between the pipette and the transfected cell, negative pressure (of max. −10 mmHg) was applied. Cells sealed with a minimum resistance of 1 GΩ were accepted for analysis. A pressure ramp from 0 mmHg to −80 mmHg with 10 mmHg increments was run (High Speed Pressure Clamp, HSPC-1, ALA Scientific Instruments) at a potential of −80 mV. Currents were digitized at 3 kHz, low-pass filtered at 1 kHz. with a patch clamp amplifier (AxoPatch 200B, Axon Instruments) and a digitizer interface (Axon Digidata 1440 A, Axon Instruments). Data analysis was performed with Clampfit 10.6 (Molecular Devices). Currents recorded at 0 mmHg pressure were taken as baseline currents (average current at 0 mmHg = −0.89 ± 0.1 pA, *n* = 385) and average and peak current during pressure pulses (500 ms) were analyzed. Initial experiments without applying a pressure ramp were conducted with a Sutter dpatch digital patch-clamp amplifier system with the SutterPatch software.

### Pull-down assay, sodium dodecyl sulfate polyacrylamide gel electrophoresis and Western blot

To check whether LecB-biotin binds to EGFP-mPiezo1, EGFP-mPiezo1-overexpressing H1299 cells were incubated with 1 µM LecB-biotin for 30 min and 3 h to allow binding, washed three times with PBS and afterwards lysed with RIPA lysis buffer at 4 °C for 30 min. Lysates were centrifuged at 15,000 × g for 20 min at 4 °C and the procedure was continued with the cleared lysates. Per 100 µL lysate, 50 µL of magnetic streptavidin-coated beads (Pierce™ Streptavidin beads, ThermoFisher Scientific) were added and incubated on an orbital shaker for 1 h at RT. Beads were separated from the lysate, which was saved for analysis, with a magnetic rack and washed three times with 25 mM TRIS and 0.05% Tween-20. The beads were resuspended in SDS loading buffer and boiled at 100 °C for 10 min to allow dissociation of the bound protein from the biotin. Lysates were saved and diluted with SDS loading buffer and boiled at 100 °C for Western Blot analysis. Lysates were centrifuged at 15,000 × g for 20 min at 4 °C and the clear supernatants were saved. Samples were loaded into a SDS gel, consisting of a 4% acrylamide stacking gel and an 8% acrylamide running gel. SDS PAGE was performed with a Bio-Rad SDS PAGE system at 110 V for 10 min and subsequently at 130 V for 70 min. Semi-dry transfer was performed with a Nitrocellulose membrane for 1 h at 0.19 A. Blocking was performed in 5% skim milk in TBS-T for 1 h. As primary antibody, mouse anti-GFP (#sc-9996, Santa Cruz) or mouse anti-Piezo1 (FAM38, MyBioSource) was incubated in a 1:1000 dilution over night at 4 °C. The membrane was washed three times with TBS-T and as a secondary antibody, horse anti-mouse IgG, HRP-linked antibody (1:1000, diluted in 5% Skim milk in 1X TBS-T; Cell Signaling, Danvers, MA, USA) was incubated for 1 h at RT. The membrane was again washed three times with TBS-T and afterwards incubated with the Novex™ ECL Chemiluminescent Substrate Reaction Kit mix (ThermoFisher Scientific). Image acquisition was performed with the ChemiDoc™ MP imaging system (Bio-Rad).

### Immunocytochemistry

5 * 10^3^ H1299 non-small lung cancer cells were seeded onto glass coverslips with a diameter of 12 mm and attachment was allowed overnight. Cells were transfected with the mPiezo1-pEGFP-N1plasmid for 48–72 h. 1 µM of fluorescently labeled LecB-Alexa Fluor 647 or unlabeled LecB was incubated on the cells for different time points. Afterwards, cells were washed three times with PBS and then fixed with 4% *para*-formaldehyde (PFA) for 10 min. Fixed cells were again washed three times with PBS. 4’,6’-Diamino-2’-phenylindole (DAPI, 1:1000) and Phalloidin-Atto 565 (1:1000) were incubated for another 30 min at RT. For Golgi staining, cells were incubated with a primary antibody against GM130 (rabbit monoclonal, ab52649, abcam, 1:100, caused slight unspecific binding in the nucleus) and subsequently with the secondary antibody goat anti-rabbit-DyLight 650, 1:200, #84546, ThermoFisher Scientific 1:200), Phalloidin-Atto 565 (1:1000) and DAPI (1:1000). Cells were again washed three times and the coverslip was mounted upside-down on a drop of Mowiol supplemented with DABCO on a glass slide. Samples were allowed to dry and were afterwards analyzed with a confocal microscope (Nikon A1R confocal microscope, 60 × oil objective, N_A_ = 1.49, used laser wavelengths: 405 nm, 488 nm, 546 nm, 647 nm) and acquired images were processed with FIJI (Version 2.14.0). For co-localization analysis, images were analyzed in FIJI with the BIOP JACoP plugin [55].

### Infection experiment with Pseudomonas aeruginosa

H1299 cells were seeded on 12 mm diameter glass coverslips and transfected with mPiezo1-pEGFP-N1as described above. The number of cells reached 48 h after transfection was estimated approx. 1 * 10^5^ cells per coverslip. One day before the experiment, pre-cultures of *Pseudomonas aeruginosa* PAO1 WT and ΔLecB strains (kindly provided by Karl-Erich Jaeger) [56] were prepared in 5 mL LB medium and incubated for 16 h at 37 °C and 180 rpm. The number of bacteria was determined via OD_600_. Bacteria were diluted in RPMI1640 to a respective MOI of 10, which corresponds to 1 * 10^6^ bacteria per well. Human cells together with bacteria were incubated for 3 h, untreated cells served as control. After incubation, the bacteria-containing medium was removed, the cells were washed three times in PBS and the cells were fixed afterwards with 16% *para*-formaldehyde for 30 min at 4 °C. Cells were again washed three times in PBS. Staining with primary antibody (anti-*Pseudomonas* (rabbit polyclonal,1:100 in 3% BSA and 0.2% Saponin in PBS)*,* #ab68538, Abcam) and secondary antibody (goat anti-rabbit-DyLight 650, 1:200, #84546, ThermoFisher Scientific) together with DAPI (1:1000) and Phalloidin-Atto 565 (1:1000) was performed and cells were mounted in Mowiol supplemented with DABCO on glass slides.

### Statistical analysis

The presented and statistically analyzed data were obtained in at least three independent experiments and are shown with the mean ± standard error of the mean (SEM). The statistical analysis is mentioned in each figure legend. Statistical tests with a p-value ≤ 0.05 were considered as statistically significant and marked by asterisks.

### Modeling

The modes of interaction between the C-terminal extracellular domain (CED, referred to as ‘cap’ region) of mechanosensitive Piezo1 channel and LecB were studied using two in silico methods. Spatial structures of proteins were taken from protein data base (PDB: 7wlu and 5a3o, respectively), missing loops were added using Modeller 9.15 [57]. Protein–protein docking of the cap trimer (aa R2214—S2454) and one LecB tetramer was performed using the ClusPro web server [58]. The top thirty docking poses were sorted, excluding those that led to potential interference with the blades of Piezo1 or the membrane.

The most probable mutual orientation of the cap and LecB, predicted by docking, was taken as initial configuration of the complex for further equilibration in the presence of explicit water and ions environment in the course of molecular dynamics (MD) simulations. The MD trajectories were calculated for the complex of the cap trimer with one LecB molecule and with three LecB molecules bound to each subunit of the cap. MD simulations were carried out using Amber22 [59] and Amber14SB force-field parameters. The protein complex was immersed into a water box with periodic boundary conditions. The TIP3P model was used for water molecules. Sodium and chloride ions were added in the required amount to neutralize the charge of the proteins and maintain the ionic strength of 150 mM. The integration step of 2 fs was used together with the SHAKE algorithm constraining the bonds involving hydrogen atoms. The Particle Mesh Ewald (PME) method was used for long-ranged electrostatic interactions. The simulations were carried out in the isotherm isobar thermodynamic ensemble at 300 K. The temperature and the pressure were kept constant using a Langevin thermostat with a collision frequency of 2 ps^−1^ and a weak coupling algorithm with a relaxation time of 2 ps, respectively. First, the system was minimized for 5000 steps and then equilibrated. In the production run, 1 μs of the trajectory were accumulated.

## Results

### LecB reduces SAC currents in wild-type epithelial cells

**Fig. 1:**
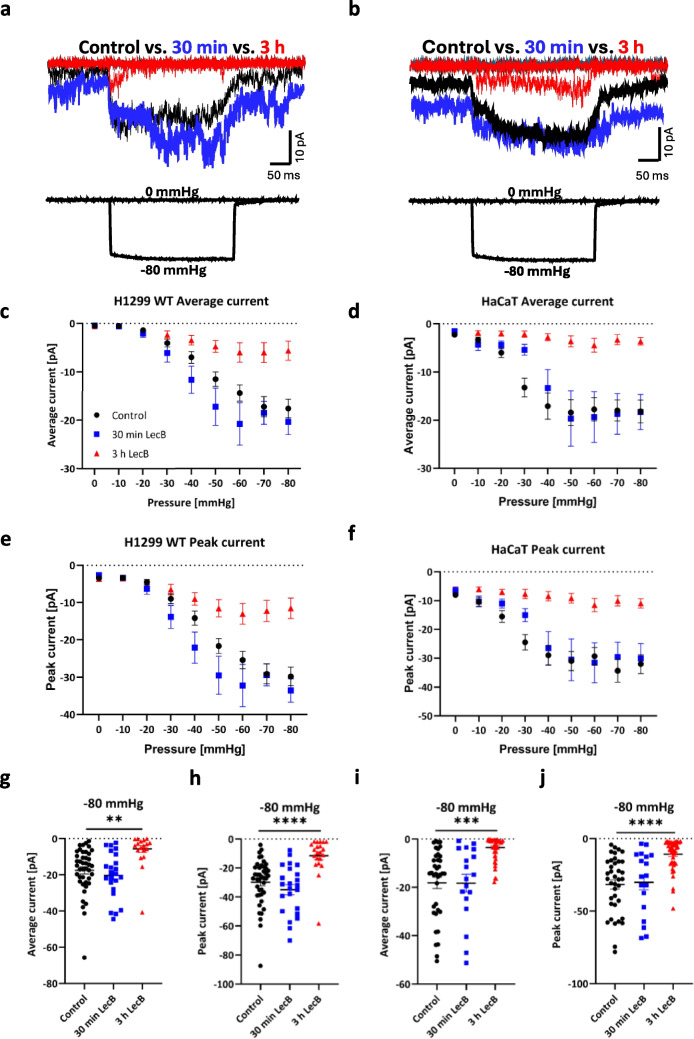
LecB modulates stretch-induced currents in H1299 non-small lung cancer cells and HaCaT keratinocytes. Mechanically induced currents were recorded by cell-attached high-speed pressure clamp in presence of LecB at different time points and compared to untreated cells. **a** and **b** depict exemplary current traces during a −80 mmHg pressure pulse lasting for 500 ms. Cells were pretreated with LecB (1 µM) for 30 min (blue traces) and 3 h (red traces) and compared to untreated cells (black traces). Quantifications of average (for H1299 cells in **c,** n _control_ = 42, n _LecB 30 min=_23–29, n _LecB 3 h_ = 21–22, and for HaCaT cells in **d**, n _control_ = 37–42, n _LecB 30 min=_ 18, n _LecB 3 h_ = 42) and peak currents (for H1299 cells in **e** and HaCaT cells in **f**) at pressure pulses from 0 to −80 mmHg with a duration of 500 ms are depicted for 30 min and 3 h of LecB treatment. Mean and SEM values are displayed. **g**, **h**, **i** and** j** depict single data points with the mean of average and peak currents at −80 mmHg pulses for H1299 cells (**g**, **h**, n _control_ = 42, n _LecB 30 min=_23, n _LecB 3 h_ = 21) and HaCaT cells (**i**, **j,** n _control_ = 37, n _LecB 30 min=_ 18, n _LecB_ _3 h_ = 42). Variations in n are due to lost seals during recordings. Significance was assessed with a one-way ANOVA and Dunnett’s multiple comparison test. * *p* ≤ 0.05; ** *p* ≤ 0.01; *** *p* ≤ 0.001; **** *p* ≤ 0.0001; ns: not statistically relevant

It has already been shown that the *P. aeruginosa* lectin LecB binds to several cell surface receptors and eventually leads to impaired physiological processes [33, 34]. To assess whether LecB has an influence on SAC, patch clamp experiments were performed in cell-attached mode. To test the effect over time, we incubated H1299 and HaCaT cells with LecB for 30 min and 3 h, respectively, and acquired ion currents in the cell-attached patch-clamp mode at a holding potential of −80 mV (Fig. S1). As *P. aeruginosa* predominantly infects wounded skin or lung tissue in immunocompromised patients, HaCaT cells and H1299 non-small lung cancer cells were chosen as cellular models. To induce stretch, a pressure protocol with pressure pulses, ranging from 0 up to −80 mmHg, in 10 mmHg increments, with a respective duration of 500 ms per pulse, was applied. Representative traces of H1299 and HaCaT cells, untreated (black) or treated with LecB for 30 min (blue) and 3 h (red) are shown in Fig. 1a (H1299) and in Fig. 1b (HaCaT).

Both untreated cell lines showed low currents at zero pressure and low-pressure pulses, resulting in low average and peak currents during the pressure steps at 0 to −20 mmHg (see Fig. 1a-d and Fig. S2−5). Increasing the pressure led to larger average and peak currents in H1299 and HaCaT cells, with average currents of −17.6 ± 1.9 pA (H1299, *n* = 42, Fig. 1c, Fig. S2) and −18.2 ± 2.4 pA (HaCaT, *n* = 37, Fig. 1d, Fig. S4) as well as peak currents of −29.8 ± 2.5 pA (H1299, *n* = 42, Fig. 1e, Fig. S3) and −32.0 ± 3.2 pA (HaCaT, *n* = 37, Fig. 1f, Fig. S5) upon pressure of −80 mmHg. These observations indicated the endogenous expression and activity of SAC in both epithelial cell types.

The potential effect of LecB on SAC in epithelial cells was evaluated for two time points. An early time point of 30 min, when LecB initially binds to the cell plasma membrane, and a later time point of 3 h after treatment, when potential cellular trafficking processes may have taken place. Cells were treated with 1 µM LecB, with respect to the tetrameric structure. For both cell lines, a 30-min LecB incubation showed no significant impact on average and peak currents at larger pressures (Fig. 1c-j, S2-5, blue data points). For example, average currents of H1299 cells after 30 min of LecB treatment were −20.3 ± 2.7 pA (*n* = 23, Fig. 1g), whereas for HaCaT cells treated for 30 min with LecB, average currents were at −18.3 ± 3.6 pA (*n* = 18, Fig. 1i). Only at −30 mmHg, average and peak currents were significantly lower for HaCaT cells treated with LecB for 30 min. For higher pressure pulses, average and peak currents did not significantly differ (Fig. 1i and j, Fig. S4 and S5). After 3 hours of Lec B incubation, currents were significantly lower in both cell types. The average current of H1299 cells was lower to −5.6 ± 2.0 pA (*n* = 21, Fig. 1g) and the peak current was lower to −11.5 ± 2.7 pA (*n* = 21, Fig. 1h) upon pressure of −80 mmHg. A similar pattern was seen for HaCaT cells: at −80 mmHg, average currents were lower after 3 h of LecB treatment (−3.6 ± 0.8 pA, *n* = 42, Fig. 1i) and the same effect was seen for peak currents (−10.9 ± 1.6 pA, *n* = 42, Fig. 1j).

For H1299 cells, a siRNA-mediated knockdown of Piezo1 was performed (Fig. S6). Observing the significantly lower stretch-induced currents in siPiezo1 cells, we conclude that Piezo1 is the dominant SAC underlying the observed activity.

Taken together, these initial results indicate the ability of LecB to modulate stretch-induced currents of epithelial cells. After 30 min of LecB incubation, we mostly observed no significant effect of LecB, with one exception (in HaCaT with -30 mmHg), whereas after 3 h of treatment, we observed that the average and peak currents both for H1299 and HaCaT cell lines were significantly lower compared to the control condition.

### LecB leads to higher Piezo1 stretch-induced currents after 30 min and to lower currents after 3 h in EGFP-mPiezo1-overexpressing H1299 cells

**Fig. 2:**
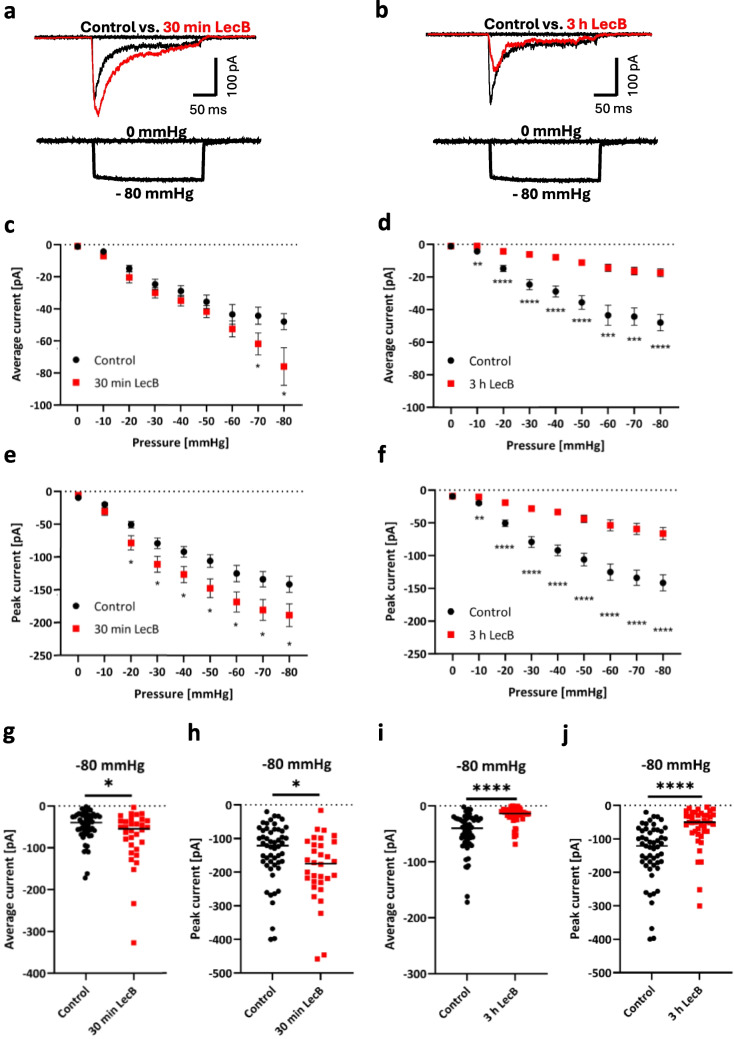
LecB modulates the stretch-induced currents of Piezo1. Mechanically induced Piezo1 currents were recorded in EGFP-mPiezo1-overexpressing H1299 cells by cell-attached high-speed pressure clamp in presence of LecB at different time points and compared to untreated cells. **a** and **b** show exemplary current traces during a −80 mmHg pressure pulse lasting for 500 ms. Cells were pretreated for 30 min (**a**) and 3 h (**b**) with LecB (1 µM, red traces) and compared to untreated cells (black traces). Quantifications of average (**c** and **d**) and peak currents (**e** and **f**) at pressure pulses from 0 to −80 mmHg with a duration of 500 ms are depicted for 30 min (**c** and **e**) and 3 h (**d** and **f**) of LecB treatment. Mean and SEM values are displayed. The values measured for the respective controls are shown in Fig. **c** as well as **d**, **e** as well as **f**, **g** as well as **i**, **h** as well as **j**, respectively. **g**, **h**, **i** and** j** depict single data points with mean of average and peak currents at −80 mmHg pulses for 30 min (**g**, **h**, n _control_ = 53, n _LecB_ = 32) and 3 h (**i**, **j,** n _control_ = 53, n _LecB_ = 42) LecB treatment. Significance was assessed with an unpaired t-test, assuming Gaussian distribution. * *p* ≤ 0.05; ** *p* ≤ 0.01; *** *p* ≤ 0.001; **** *p* ≤ 0.0001; ns: not statistically relevant

Based on lower SAC currents in H1299 and HaCaT wild-type cells for the 3-h LecB treatment and the evidence from a mass spectrometry analysis of the LecB interaction partners, we wanted to investigate specifically Piezo1 channel currents upon stretch in the presence of LecB. We incubated EGFP-mPiezo1-overexpressing H1299 cells with LecB for 30 min and 3 h, respectively, and acquired ion currents in the cell-attached patch-clamp mode with the identical pressure protocol used for WT cells. Representative traces of the respective conditions at −80 mmHg are depicted in Fig. 2a (for 30 min) and Fig. 2b (for 3 h). A higher mechanically-induced Piezo1 current was observed after 30 min of LecB treatment (red trace) compared to untreated cells (further stated as control, black trace) (Fig. 2a), while the current was much lower for 3 h of LecB treatment compared to control (Fig. 2b). In the following, the acquired results are depicted in more detail as average currents (Fig. 2c and d) and peak currents (Fig. 2e and f) at pressure pulses varying from 0 mmHg to −80 mmHg. The typical stretch-dependent increase of the Piezo1 current is depicted by the black traces. Interestingly, we detected higher currents with LecB treatment for 30 min (Fig. 2c and e), suggesting that LecB is enhancing stretch-dependent currents. For example, peak currents were already significantly higher for 30 min of LecB treatment upon −40 mmHg pressure pulses. Both average and peak currents were significantly higher for cells treated with LecB for 30 min at higher pressure pulses, e. g. at −80 mmHg compared to control. The control cells displayed a mean average current of −48.0 ± 5.0 pA (*n* = 53), whereas cells treated with LecB for 30 min showed a mean average current of −75.9 ± 11.7 pA (*n* = 32) (Fig. 2g). Similar effects were seen for peak currents (Fig. 2h): The control cells showed mean peak currents of −141.5 ± 12.2 pA (*n* = 53) at −80 mmHg, which are significantly lower than the mean peak currents of −188.6 ± 17.2 pA (*n* = 32) for cells treated with LecB for 30 min. The complete measurement series for 30 min of LecB treatment are displayed in Fig. S7 (average currents) and Fig. S8 (peak currents) for each applied pressure pulse (ranging from 0 mmHg to −80 mmHg). In summary, these results highlight that a treatment of cells with LecB for 30 min induces higher stretch-induced channel currents of Piezo1.

Since it has already been shown that LecB is partially internalized in the first hours after binding to the plasma membrane of epithelial cells [33, 60], we wanted to investigate whether LecB is able to modulate the currents of Piezo1 also at later incubation times (i.e. at 3 h). Therefore, we performed the same protocol after 3 h of incubation with LecB (1 µM). The quantifications of average and peak currents are depicted in Fig. 2d and 2f. In general, Piezo1 stretch-induced currents were lower after 3 h of LecB treatment compared to control cells. Already at pressure pulses of −10 mmHg, we measured significantly lower average currents (−4.3 ± 0.7 pA, *n* = 59 patched cells for control, vs. −1.8 ± 0.5 pA, *n* = 42 cells for 3 h of LecB treatment) as well as peak currents (−19.7 ± 2.4 pA, *n* = 59 cells for control, vs. −10.4 ± 2.0 pA, *n* = 42 cells for 3 h of LecB treatment). This effect was also well observed at pressure pulses of −80 mmHg and showed again a significant decrease of average currents (Fig. 2i, −48.0 ± 5.0 pA, *n* = 53 patched cells for control, vs. −17.5 ± 2.4 pA, *n* = 42 cells for the 3-h LecB treatment) and peak currents (Fig. 2j, −141.5 ± 12.2 pA, *n* = 53 cells for control, vs. −66.0 ±9.5 pA, *n* = 42 cells for the 3-h LecB treatment). The complete measurement series for 3 h of LecB treatment are displayed in Fig. S9 (average currents) and Fig. S10 (peak currents) for each applied pressure pulse (ranging from 0 mmHg to −80 mmHg). It is important to note that LecB treatment did not completely suppress the channel activity of Piezo1, but rather lowered the average and peak currents.

Recordings of EGFP-overexpressing H1299 cells, which express hPiezo1 endogenously, were also acquired. In general, the average currents of the EGFP-transfected cells were lower compared to EGFP-mPiezo1-overexpressing cells (Fig. S11). The decrease in Piezo1 currents after 3 h of LecB incubation compared to untreated cells (control) was quite clear for all applied pressure pulses, while the enhancing effects observed in EGFP-mPiezo1-overexpressing cells at 30 min were not observed (Fig. S11).

In summary, LecB was slightly enhancing the stretch-induced currents of EGFP-mPiezo1-overexpressing cells at early stages of incubation while it was strongly decreasing Piezo1 currents at later stages.

### The carbohydrate binding ability of LecB is essential for modulating Piezo1 currents in EGFP-mPiezo1-overexpressing H1299 cells

After we could confirm the LecB-triggered reduction of stretch-induced currents in H1299 cells that were transiently transfected with an EGFP-mPiezo1 construct, we wanted to assess, whether the competitive blocking of the carbohydrate-binding pocket of LecB with l-Fucose could affect the observed effects. We added 45 mM l-fucose and 10 mM para-nitrophenyl-galactopyranoside (PNPG), an inhibitor of the *P. aeruginosa* lectin LecA [33, 61]. We could observe that the incubation of l-fucose together with LecB prevented the latter from reducing stretch-activated currents after 3 h. For example at.

**Fig. 3:**
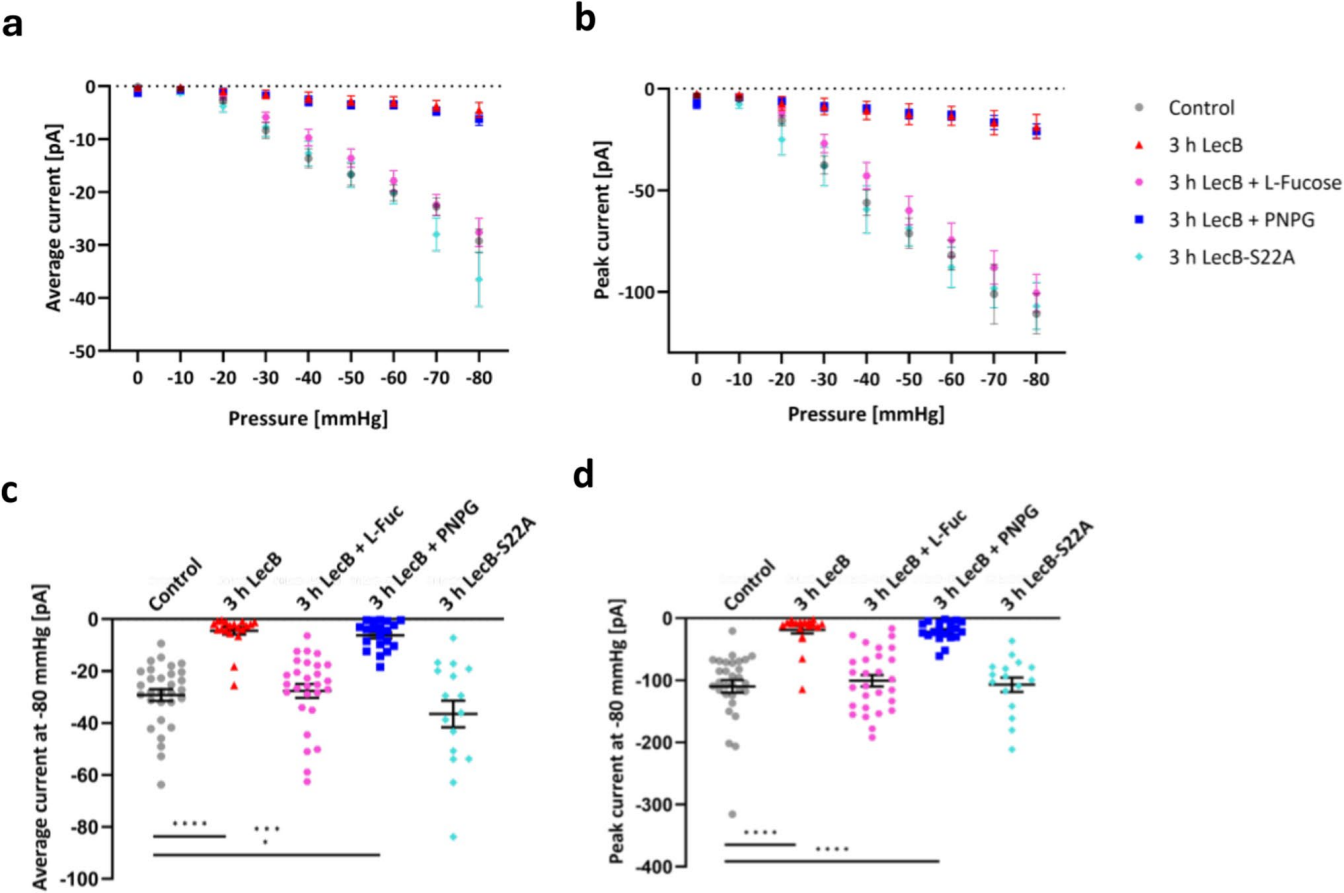
The carbohydrate binding ability of LecB is crucial for affecting Piezo1 currents. Mechanically induced Piezo1 currents were recorded in EGFP-mPiezo1-overexpressing H1299 cells by cell-attached high-speed pressure clamp in presence of LecB alone and with different inhibitors, and the carbohydrate-binding site mutant LecB-S22A for 3 h, compared to untreated cells. **a** and **b** depict average and peak currents for untreated (black), and LecB- (red), LecB + l-fucose- (green), LecB + PNPG- (purple) and LecB-S22A- (blue) treated cells at pressure pulses from 0 to −80 mmHg. n _control_ = 30, n _LecB_ = 20, n _LecB+L-Fuc_ = 28, n_LecB_ _+PNPG_ = 20, n _LecBS22A_ = 16. **c** and **d** show plots of average and peak currents at pressure pulses from 0 to −80 mmHg with a duration of 500 ms for 30 min and 3 h of LecB treatment and additional inhibitors, and the mutant LecB-S22A. Mean and SEM values are displayed. Significance was assessed with a one-way ANOVA with Dunnett’s multiple comparison test. * *p* ≤ 0.05; ** *p* ≤ 0.01; *** *p* ≤ 0.001; **** *p* ≤ 0.0001; ns: not statistically relevant

−80 mmHg, we recorded similar and not significantly varying average currents (−29.2 ± 2.2 pA for control cells, *n* = 30, vs. −27.6 ± 2.7 pA for LecB + l-fucose-treated cells, *n* = 28) and peak currents (−110.8 ± 9.9 pA for control cells, *n* = 30, vs. −100.6 ± 9.2 pA for LecB + l-fucose-treated cells, *n* = 28), compared to control cells (Fig. 3a-d for single data points at −80 mmHg pulses). On the other hand, the addition of PNPG to LecB was as expected not able to rescue stretch-induced currents (average currents at −80 mmHg: −110.8 ± 9.9 pA for control cells, *n* = 30, vs. −6.2 ± 1.2 pA for LecB + PNPG-treated cells, *n* = 20). The average and peak currents were as high as in cells that were treated only with LecB for 3 h (−6.2 ± 1.2 pA for LecB + PNPG-treated cells, *n* = 20, vs. −4.5 ± 1.4 pA for LecB-treated cells, *n* = 20, Fig. 3a-d).

Additionally, we incubated EGFP-mPiezo1-overexpressing H1299 cells with a LecB mutant, in which the serine at position 22 was mutated to an alanine (LecB-S22A) [49]. This point mutation is located in the carbohydrate-binding pocket of LecB and prevents LecB from binding to l-fucose or fucose-containing oligosaccharides, whereas D-mannose can still bind [49]. Interestingly, average and peak currents after 3 h of LecB-S22A treatment were not significantly changed compared to untreated control cells (average currents at −80 mmHg: −36.5 ± 5.1 pA for LecB-S22A-treated cells, *n* = 16, vs. −110.8 ± 9.9 pA for control cells, *n* = 30), indicating that the ability of LecB to bind to l-fucose-containing glycans is crucial for affecting stretch-activated currents, and especially Piezo1 currents, in epithelial cells (Fig. 3a-d). To summarize, these findings indicate that LecB reduces SAC currents through its binding to fucosylated receptors at the plasma membrane.

### LecB has an impact on the abundance and distribution of mPiezo1 at the plasma membrane

**Fig. 4:**
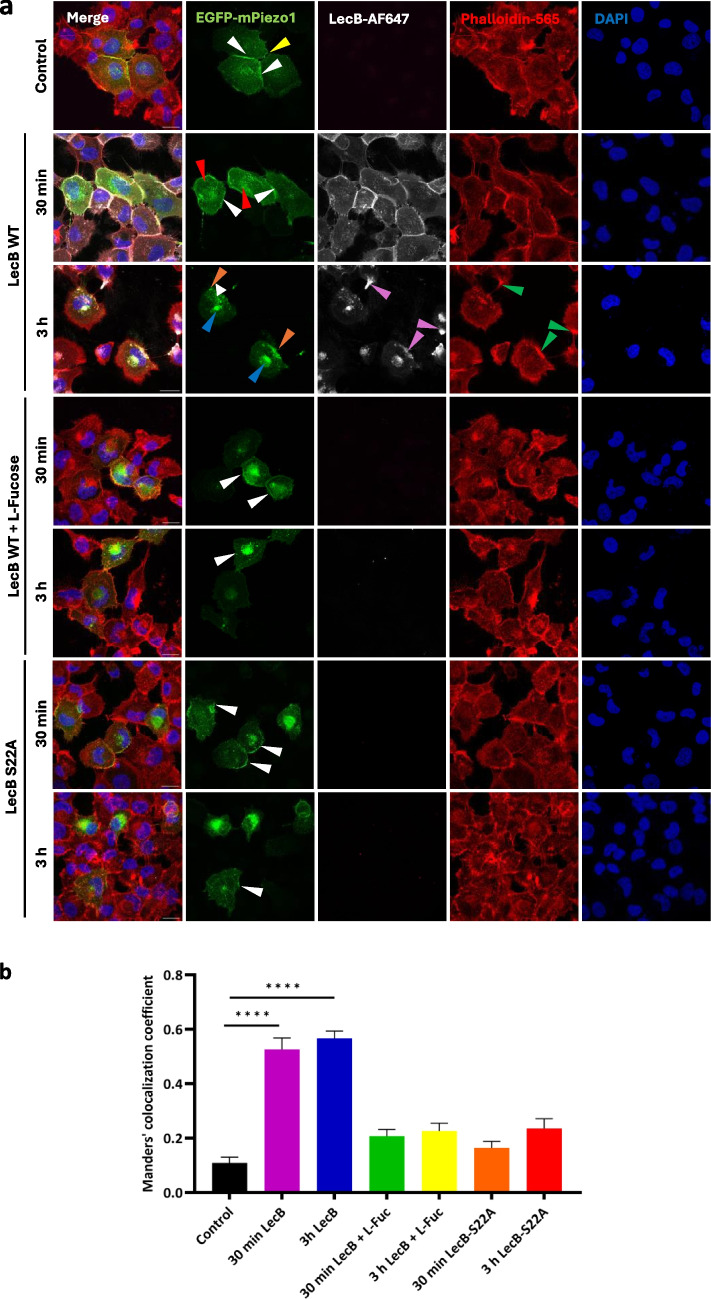
LecB internalization and relocalization depend on its binding to fucosylated structures. (**a**) EGFP-mPiezo1-overexpressing H1299 cells were treated either with LecB-Alexa Fluor 647 (shortly LecB-AF647, 1 µM), LecB-AF647 (1 µM) in combination with l-fucose (45 mM) or LecB-S22A-AF647 (1 µM) for 30 min and 3 h at 37 °C, respectively. Cells that were not treated with LecB served as control. After LecB treatment, cells were fixed with 4% PFA. For the staining of actin cytoskeleton and nuclei, cells were permeabilized with a PBS solution containing 0.2% Saponin and 3% BSA and then treated with Phalloidin-Atto 565 (shortly Phalloidin-565) and DAPI, respectively. The presented images were acquired by confocal microscopy, and Z projections are shown. White and yellow arrowheads indicate the physiological distribution of EGFP-mPiezo1 without LecB, red arrowheads indicate partial overlapping of EGFP-mPiezo1 and LecB-AF647 in endosomes after 30 min of LecB-AF647 treatment, and orange arrowheads indicate EGFP-mPiezo1 relocalization. Orange arrowheads point to the co-localization of LecB with EGFP-mPiezo1 at the posterior part of the cell, whereas blue arrowheads point to the co-localization of LecB with EGFP-mPiezo1 in the perinuclear area. Pink arrowheads point to the accumulation of LecB at the posterior part of the cell. Green arrowheads indicate accumulation of F-actin at the posterior parts of the cell. Scale bars: 20 µm. (**b**) Co-localization analysis of EGFP-mPiezo1 with LecB-AF647 or LecBS22A-AF647. The Manders’ co-localization coefficient was assessed for the respective conditions shown in (a). Data was statistically analyzed with one-way ANOVA and Dunnett’s multiple comparison test, Dunnett’s

Since LecB affected Piezo1 currents, we next investigated the cellular localization of the two proteins and a possible co-localization over the time course of 3 h. Therefore, EGFP-mPiezo1-overexpressing H1299 cells were incubated at 37 °C with 1 µM of Alexa Fluor 647-labeled LecB (from now on called LecB-AF647) for 30 min and 3 h, respectively. We furthermore added l-fucose to the LecB treatment and, as a third condition, we incubated the cells with 1 µM of the binding site mutant LecB-S22A-AF647. Cells were fixed and stained with Phalloidin-Atto 565 and DAPI for the visualization of the F-actin cytoskeleton and the nuclei for easier orientation. In the absence of LecB, EGFP-mPiezo1 was mainly located at the plasma membrane, but was unevenly distributed; the main sites appeared to be cell–cell contacts (Fig. 4a, white arrowheads) and lamellipodia Fig. 4a, yellow arrowhead). After 30 min of incubation, LecB-AF647 was still present at plasma membrane, but also partially internalized. By assessment of the Manders’ co-localization coefficient (MCC) between EGFP-mPiezo1 and LecB-AF647, we could detect increasing co-localization over time (Fig. 4b) both at intracellular structures, probably diverse endosomes (Fig. 4a, 30 min, red arrowheads), and at the plasma membrane, especially at cell–cell contacts (Fig. 4a, 30 min, white arrowheads). After 3 h of incubation, the appearance of LecB-AF647 changed dramatically compared to the 30 min time point. In addition to its increased accumulation in the perinuclear region (Fig. 4a, 3 hours, blue arrowheads), LecB-AF647 appeared to be concentrated in the posterior part of the cells both in non-transfected cells and in EGFP-mPiezo1-overexpressing cells (Fig. 4a, 3 hours, pink arrowheads), which coincided with a strong re-localization and local accumulation of EGFP-mPiezo1 at sites of LecB-AF647 (Fig. 4a, 3 hours, orange arrowheads). Furthermore, F-actin also accumulated at these sites (green arrowheads).

As in the electrophysiological experiments, we also tested whether LecB-AF647 incubated in combination with l-fucose, or the mutant LecB-S22A-AF647 co-localized with EGFP-mPiezo1. The observation of the representative images in Fig. 4a and the quantification of MCC show a much lower binding to the cells in general. Fluorescent signal could not be seen by eye when the cells of different conditions were acquired with identical laser settings and intensities, but some small amounts of WT LecB (not neutralized by l-fucose) and mutant LecB must have bound to the cells as the respective MCC were slightly higher compared to untreated control cells (Fig. 4b). Nevertheless, the MCC of the cells that were treated with the WT LecB was significantly higher than the MCCs of LecB + l-fucose or LecB-S22A with EGFP-mPiezo1. Furthermore, the intensities of LecB-AF647 treated cells for 30 min and 3 h was significantly higher compared to untreated control cells, whereas intensities for LecB-AF647 + l-fucose as well as LecB-S22A-AF647-treated cells were rather similar to control cells (Fig. S12). This clearly indicates that the functional, fucose-binding carbohydrate binding pocket is crucial for the binding of LecB to the cells and the subsequent relocalization of EGFP-mPiezo1. Concluding these observations, LecB seems to be able to form a distinct domain consisting of accumulated EGFP-mPiezo1 and F-actin, located at the posterior part of cells.

**Fig. 5:**
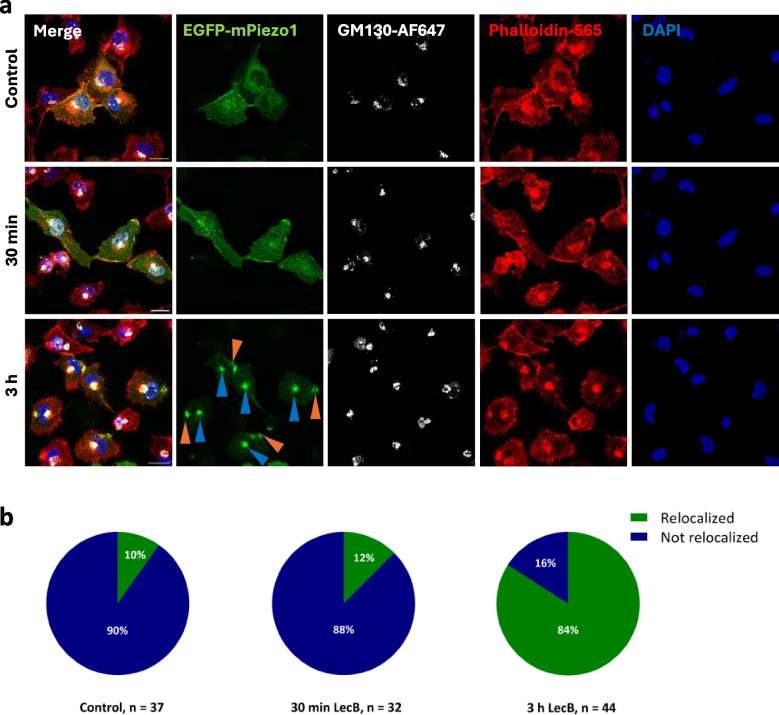
LecB-AF647 and EGFP-mPiezo1 accumulate at the posterior part of the cell. (**a**) EGFP-mPiezo1-overexpressing H1299 cells were treated with LecB-AF647 (1 µM) for 30 min and 3 h at 37 °C, respectively. Cells that were not treated with LecB served as control. After treatment, cells were fixed with 4% PFA. For Golgi staining, cells were permeabilized with a PBS solution containing 0.2% Saponin and 3% BSA and then treated with an anti-GM130 antibody and subsequently with the respective secondary antibody, linked to AlexaFluor-647, Phalloidin-Atto 565 and DAPI, respectively. Orange arrowheads point to the co-localization of LecB with EGFP-mPiezo1 at the posterior part of the cell, whereas blue arrowheads point to the co-localization of LecB with EGFP-mPiezo1 in the perinuclear area. The depicted images were acquired by confocal microscopy and Z projections are shown. Scale bars: 20 µm. (**b**) Pie charts showing the re-localization of EGFP-mPiezo1 to the cell’s posterior part in control cells and cells treated with LecB-AF647 for 30 min and 3 h. Green parts indicate the re-localization of EGFP-mPiezo1, whereas blue parts represent cells in which no accumulation at the posterior part could be observed

To analyze in more detail, whether EGFP-mPiezo1 relocalizes due to LecB to the posterior part (i.e. “the rear of the migrating cell”), and to a certain extent also to the perinuclear area of a cell, we stained the cells additionally with the Golgi marker GM130, in addition Phalloidin-Atto 565 to illustrate cell borders. By assessing the position of the Golgi apparatus with regard to the nucleus, it is possible to identify the migrating, i.e. leading edge of a cell, and accordingly also the cell rear. This effect was seen in various migrating cells that display the so-called front-rear polarity [62, 63]. To quantify the LecB-induced relocalization of EGFP-mPiezo1, the intensity profiles of EGFP-mPiezo1 were measured by a line scan along the front-rear axis of a cell (Fig. S13). In untreated cells (90%) or cells treated with LecB for 30 min (88%), EGFP-mPiezo1 was widely distributed throughout the cell, e.g. at cell borders, in endosomal structures, and in the perinuclear area, but without significant accumulation at any specific location within the cell that we could detect. However, in cells treated with LecB for 3 h, we observed a different distribution with accumulations in the perinuclear area, overlapping with the GM130 signal (Fig. 5a, 3 hours, blue arrowheads), and at the cell rear (Fig. 5a, 3 hours, orange arrowheads), which was considered to be a relocalization of mPiezo1. This relocalization at the cell rear and the perinuclear area was observed in approximately 84% of the EGFP-mPiezo1-positive cells that were incubated with LecB for 3 h. In comparison, this effect was only observed in about 10% of untreated cells and 12% of the cells treated with LecB for 30 min, also depending on the cell density.

To assess whether the relocalization of EGFP-mPiezo1 can be observed upon bacterial infection, we incubated EGFP-mPiezo1-overexpressing H1299 cells with *P. aeruginosa* PAO1 wild-type (WT) and ΔLecB strains with an MOI of 10 for 3 h and performed immunofluorescence staining with an anti-*Pseudomonas* antibody. We could detect changes in the intracellular distribution of EGFP-mPiezo1, but a clear relocalization of EGFP-mPiezo1 to the posterior part of the cell (Fig. S14), as seen for cells treated with LecB-AF647, was not evident. It is important to mention that the Piezo1-positive structures, which formed upon bacterial infection, were also not clearly co-localizing with stained bacteria. In cells infected with the *P. aeruginosa ΔLecB* strain, the EGFP-mPiezo1 distribution resembles rather to uninfected cells than cells that were infected with the wild-type *P. aeruginosa* strain (Fig. S14). The absence of LecB in the *P. aeruginosa* PAO1 *ΔLecB* strain was confirmed via Western Blot of bacterial lysates and antibody staining with an antibody against LecB (Fig. S15).

Our data from confocal microscopy indicate that LecB alters the abundance of Piezo1 at the plasma membrane within the first 3 h. In particular, we observed a LecB-induced translocation of Piezo1 to the posterior of the cells, clearly visible after 3 h, which potentially affects its channel activity.

### LecB interacts with Piezo1 in a carbohydrate-dependent manner particularly evident after 3 h

Next, we wanted to investigate possible molecular interactions between Piezo1 and LecB responsible for the observed activation and co-localization. Since immunoprecipitation would have been challenging for this particular method due to the limited quality of available antibodies for both LecB and Piezo1, we decided to perform pull-down experiments with biotinylated LecB (LecB-biotin) after lysis of EGFP-mPiezo1-overexpressing H1299 cells. We confirmed the presence of Piezo1 biochemically by immunoblotting.

**Fig. 6:**
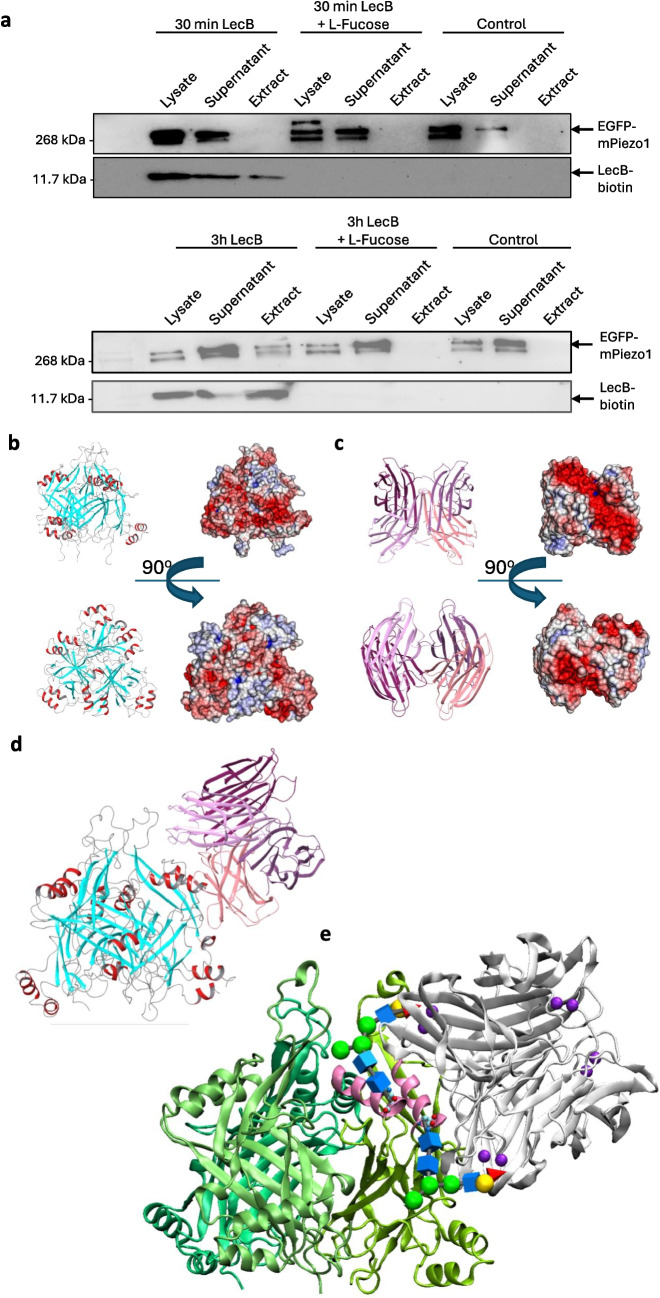
Carbohydrate-mediated interactions of LecB and Piezo1. EGFP-mPiezo1-overexpressing H1299 cells were treated with LecB-biotin (1 µM) for 30 min and 3 h, respectively, or remained untreated. To block LecB binding to glycosylated receptors at the plasma membrane, l-fucose (45 mM) was partly applied. After lysis, cell lysates were incubated with magnetic streptavidin beads. Lysate, supernatant and extract were run over an 8% acrylamide SDS PAGE. NC membranes were stained with an anti-GFP primary antibody followed by an anti-mouse horseradish peroxidase secondary antibody. M = Marker. **a** and **b**: Immunoblots depict EGFP-mPiezo1 signals of different strength respective to the applied treatment for 30 min (**a**) and 3 h (**b**). Figures c-d show potential interactions between LecB and the Piezo1 cap domain based on electrostatic potentials. (**c**) The trimeric cap region of Piezo1 is displayed in cartoon presentation (β-sheets are shown in cyan and α-helices in red) in side view and top view. The distribution of the electrostatic potential from negative to positive values is shown as a color gradient from red to blue (generated with eF-surf; available online: https://pdbj.org/eF-surf/top.do). (**d**) LecB homo-tetramer with color differentiation of subunits, in top view and side view. (**e**) Complex of the cap region of Piezo1 with LecB equilibrated in the course of MD trajectories. Figure **f** shows a putative interaction based on Piezo1 glycosylation. The binding of the tetrameric LecB to 1,4-fucose residues of a putative assembly of two complex N-glycans containing the Le^a^ epitope on the surface of the trimeric cap region of Piezo1 is shown. The Ca^2+^-atoms in the carbohydrate binding sites are displayed as purple spheres. The helices bearing the two N-glycosylation sites are highlighted in pink. N-glycans are presented according to SNFG nomenclature (GlcNAc – blue rectangular, Man – green sphere, Gal – yellow sphere, Fuc – red triangle). Glycosylated residues N2310 and N2347 are shown as spheres with cyan for carbon, red for oxygen and blue for nitrogen atoms. The position of Le.^a^ epitopes in the carbohydrate binding sites of LecB corresponds to the known modes of interactions (PDB: 4UT5)

After pull-down of LecB-biotin from the lysate fraction with magnetic streptavidin beads (extract fraction) and immunoblotting against anti-GFP, EGFP-mPiezo1 was clearly identified as a LecB interacting partner for the 3-h time point, whereas after 30 min, no interaction could be detected yet (Fig. 6a). Piezo1 signals were also observed in the supernatant fraction, indicating that not all Piezo1 molecules interact with LecB. In the absence of LecB, no Piezo1 signal could be detected in the extract fraction, whereas an expected signal was present in the supernatant fraction (Fig. 6a). Notably, LecB-biotin detection via an anti-LecB antibody was confirmed for cells that were treated with LecB-biotin, whereas LecB-biotin treatment in combination with l-fucose prevented the LecB-biotin to bind to fucosylated plasma membrane receptors and was therefore not detected in the lysate, supernatant and extract. It is important to mention, that the estimated and observed molecular weight of LecB is 11.7 kDa. However, as we wanted to detect EGFP-mPiezo1 and LecB-biotin signals on the same Western Blot membranes, we used an 8% acrylamide SDS gel. With this pore size, the protein bands at low molecular weights (≤ 30 kDa), were not separated properly, which is why the LecB band was seen at the same height as the 31 kDa ladder (Fig. S16). To prove that LecB-biotin is not binding unspecifically to proteins, we performed Ponceau staining that shows faint and less bands in the extract conditions (Fig. S16).

It was shown that LecB has one carbohydrate binding site per monomer with a high affinity for l-fucose [31] and thus targets glycosylated host cell receptors, such as integrins [33]. By saturating the carbohydrate binding sites of LecB with l-fucose, we successfully blocked the binding of LecB to plasma membrane receptors in various epithelial cells [32, 33]. Using the same strategy, we pre-incubated 1 µM of LecB-biotin with 45 mM l-fucose to saturate the carbohydrate binding sites of LecB-biotin. We then incubated the complexes with EGFP-mPiezo1-overexpressing H1299 cells for 30 min and 3 h, followed by pull-down assays. Interestingly, we observed no Piezo1 signal in the extracts of those conditions treated with LecB-biotin in combination with l-fucose (Fig. 6a), indicating that l-fucose efficiently blocked an interaction.

It remains to be clarified whether these interactions between LecB and Piezo1 occur directly via protein–protein or protein-carbohydrate interactions or indirectly via interactions with other molecules that are glycosylated and belong to a potential LecB-induced membrane domain.

### In silico analysis reveals a preferred orientation of LecB in respect to the Piezo1 cap region and the N-glycosylation sites

Piezo1 has several potential glycosylation sites, i.e. in the cap region and in the blades, but little is known about the nature of the oligosaccharide chains [64]. To predict and get insight into potential modes of interactions between the cap region of Piezo1 and LecB at atomistic level, computer modelling was performed. The distribution of the electrostatic potential around the two protein molecules (Fig. 6b for Piezo1 cap region and Fig. 6c for LecB) implies the existence of their preferred mutual orientations. The protein–protein docking results confirm that a positively charged patch of Piezo1 and a negatively charged patch of LecB are involved in the interactions. After discarding the docking poses that would interfere with the membrane, only one binding orientation between the cap of Piezo1 and LecB remained. In this complex, two helices of Piezo1 (aa S2300-N2310 and N2347-L2358; numbering as in uniprot E2JF22) are in contact with part of the groove formed in the LecB tetramer. Such a complex can be formed by any of the three subunits of the cap, resulting in the binding of at least three LecB tetramers (Fig. S17). The orientation of the helices of the cap and the spatial dimensions do not allow the LecB molecules to interfere with each other (Fig. S17). Molecular dynamics simulations showed that the complexes with one LecB molecule as well as with three LecB molecules remain stable in the course of 1 μs trajectories (Fig. 6d and Fig. S17). The contacts between the cap region of Piezo1 and LecB are formed by charged and polar residues (see complete list in supplementary Table T1).

The helices S2300-N2310 and N2347-L2358 of the cap region of Piezo1 contain two amino acids that can be glycosylated. The presence of N-glycans covalently bound to N2310 and N2347 has been demonstrated for human Piezo1 [64]. Our experimental studies (Fig. 6a) revealed that glycans play a crucial role in the binding of LecB. Therefore, in a next step, we investigated how the carbohydrate-binding site of LecB could accommodate a putative l-fucose-containing N-glycan from the cap region of Piezo1, where electrostatic potentials also favor interactions. To the best of our knowledge, there is no information on the exact sequence of the glycans of Piezo1. A glycan array showed that LecB from *P. aeruginosa* strain PAO1 has a major preference to SLe^a^, Le^a^ and H-Type2 oligosaccharides [65]. In addition, an available X-ray structure illustrates an interaction mode between Le^a^ and LecB [65]. Using this information, we reconstructed a generalized structure of the complex glycan and conjugated it to both N2310 and N2347 residues of the cap region of Piezo1. The complex N-glycan was formed by attaching the Le^a^ blood group antigen to the 1,6-mannose arm of the core glycan. The glycans exhibited an extended conformation to determine a minimum N-glycan chain length that simultaneously fits two LecB binding sites. The length of the two glycans was six sugar residues, which corresponds to around 20 Å. Both glycans perfectly match the carbohydrate binding sites of LecB when bound to the cap of Piezo1 (Fig. 6e), with the Le^a^ epitope in perfect overlay with the position known from X-ray structure [65].

## Discussion

The main goal of this study was to investigate, whether *P. aeruginosa* LecB is able to modulate Piezo1 SAC activity at the plasma membrane and/or localization, which could have a drastic impact on host cell processes and help bacterial infection. In this study, we focused on the stretch-activated, non-selective cation channel Piezo1, which emerged as a potential LecB interaction partner in a mass spectrometry analysis.

### LecB effects on Piezo1 currents and localization

Based on cell-attached patch clamp experiments in wild-type H1299 and HaCaT cells, we demonstrated no significant change in SAC activity for H1299 and HaCaT cells that were treated with LecB for 30 min, respectively. To investigate this observation further, we used EGFP-mPiezo1-overexpressing H1299 cells to evaluate stretch-induced Piezo1 currents after 30 min of LecB incubation. We could detect slight but significantly larger stretch-activated Piezo1 currents, as reflected by both higher average and peak currents (for pressure −70 to −20 mmHg, respectively) compared to control. Under the same experimental conditions, we observed by fluorescence microscopy that LecB partially co-localized with Piezo1 at the plasma membrane, most pronounced at cell–cell contacts, and also began to internalize to some extent. Regarding the preference of LecB for cell–cell contacts, it was previously shown, using giant unilamellar vesicles as membrane models, that LecB is able to crosslink opposing membranes by binding and reorganizing glycolipids [66]. Concerning the localization of Piezo1, it has been recently demonstrated that Piezo1 can be directed to cell–cell junctions by PECAM1 and CDH5 [43]. Under pathological conditions, the tetravalent LecB could play an important role in the recruitment and translocation of these molecules, thereby modulating force sensing.

By binding to glycolipids, LecB can cause negative membrane curvature, which leads to the formation of tubular membrane invaginations in giant unilamellar vesicles [66]. For its uptake into epithelial cells, the tetravalent LecB probably uses a similar strategy, namely the crosslinking of glycosphingolipids and glycosylated receptors, such as integrins, to generate inward-facing membrane tubules [33]. It is also known that Piezo1 negatively curves the cell plasma membrane and that membrane flattening through mechanical tension leads to its activation [54, 84, 85]. LecB can potentially change the local membrane curvature and thus may influence the membrane integration of Piezo1 and its activity during the 30 min treatment. However, only slightly higher currents have been observed in H1299 cells treated with LecB for 30 min, an effect that is more pronounced for EGFP-mPiezo1-overexpressing cells.

After the longer LecB incubation of 3 h, the opposite effect was observed, in particular clearly lower Piezo1 currents upon stretch compared to the untreated condition. This effect was confirmed both in wild-type cells and in EGFP-mPiezo1-overexpressing cells, indicating a (patho-)physiological relevance. The current-reducing effect on EGFP-mPiezo1-overexpressing H1299 cells after 3 h of LecB treatment was fully dependent on the carbohydrate-binding pocket of LecB. Since LecB has been shown in the past to interact with and internalize several host cell surface receptors and extracellular matrix proteins in different cell types, such as IGF1R, β1-Integrin, α3-integrin, and laminin [32–34], our first assumption was that Piezo1 should be internalized by LecB. Based on fluorescence microscopy images, co-localization analysis, and pull-down results, we conclude that LecB and Piezo1 were still mostly together after 3 h of LecB incubation. Both proteins were partly internalized and transported to the perinuclear area, while the most pronounced co-localization and signal were observed at the posterior of the cells. To determine the location of the posterior part of the cell, we stained the Golgi apparatus with an anti-GM130 antibody. Yadav and colleagues described the positioning of the Golgi apparatus towards the leading edge of front-to-rear-polarized migrating cells, and in particular in front of the nucleus [62, 63]. Based on the staining of the Golgi apparatus, we could clearly detect a LecB-mediated relocalization of EGFP-mPiezo1 towards the posterior part of the cells. Moreover, this observation was dependent on the intact carbohydrate-binding pocket of LecB, as LecB in co-incubation with l-fucose and the mutated LecB-S22A did not lead to EGFP-mPiezo1 relocalization. Compared to the 30-min time-point and the untreated control, EGFP-mPiezo1 localization changed drastically, potentially explaining lower currents in the presence of LecB as most channels localize at the rear of the cells, away from the region close to the nucleus used to record channel activities. Future work will explore whether translocated Piezo1 channels are conductive at the plasma membrane or internalized.

Piezo1 plays an important role in cell coordination and migration, e. g. in keratinocytes [67], but also in angiogenesis in cancer cells [40, 81] and microglia [68]. Its chemical activation led to increased cell migration of stem cells and its physical activation to regulated migration in keratinocytes [44]. Holt and colleagues described that Piezo1 accumulated at the edges of wounds in a mouse wound model and that Piezo1 was found at the rear of cells in a single cell model, both of which are associated with impaired wound healing [44].

LecB also has a dramatic effect on wound healing by blocking cell migration, as has been shown using in vitro [33] and in vivo [36] models. Cott et al. suggested that LecB has an antagonizing effect of the canonical Wnt signalling pathway via GSK-3β, resulting in β-catenin degradation, thus affecting the regulation of cell proliferation [32]. This also affects the cell’s ability to migrate and maintain physiological front-rear polarity, as β-catenin has been shown to be crucial for directional cell migration and polarity [69], and to be associated with γ-tubulin, a centrosome marker, upon LecB treatment. Thuenauer et al. could confirm that upon LecB treatment of epithelial cells, other important keyplayers of cell migration and adhesion, such as β1-integrin, are internalized and degraded [33]. Cott et al. showed a strong co-localization between β-catenin and β1-integrin upon LecB treatment, in particular intracellularly and at the centrosome [32]. Interestingly, our findings on a relocalization of Piezo1 to the posterior part of the cell lead to the same direction, as we used the orientation of the Golgi apparatus towards the nucleus to localize the posterior part of the cell. Cott et al. investigated co-localization between β-catenin and γ-tubulin, which marks the centrosome. As centrosomes were long stated being localized at the front, or leading edge of a cell, Zhang and Wang stated the centrosome being localized at the rear of the cell in mesenchymal migration [70]. Since the H1299 cells are originally epithelial cells, that show characteristics of the mesenchymal type [71], we conclude that the centrosome is also located at the posterior part of the H1299 cells. Therefore, we hypothesize that LecB is able to induce a membrane domain at the posterior part of the cell, which may not only consist of Piezo1 and F-actin, but also β1-integrin, β-catenin, and potential other host cell molecules, causing the strong effects on wound healing and cell proliferation which were shown previously [32–34]. Future work needs to focus on this topic, in particular on whether this hypothesis can be confirmed and observed in other cell types.

By confirming that LecB has a strong inhibitory effect on SAC, and especially on Piezo1, we add another dimension to the broad spectrum of effects of LecB on epithelial cells. Piezo1 mediates the influx of Ca^2+^, an important second messenger for various cellular physiological processes. For example, Ca^2+^ triggers the activity of numerous proteases, such as the members of the disintegrin and metalloproteinase (ADAM) family, in particular ADAM10 [72]. ADAM10 is an upstream member of the Notch signaling pathway and is able to cleave its substrates and thereby activate them. One important substrate is Notch1, a regulator for inflammatory processes, cell migration and epithelial-to-mesenchymal transition [73, 86, 87]. By cleaving Notch1 through ADAM10 and subsequently γ-secretase to the transcription factor Notch1 intracellular domain, inflammation is suppressed and cell migration is attenuated [73, 86]. As previous studies show exactly these phenotypical effects of LecB, namely increased inflammation and impaired cell migration, LecB could possibly also target this signaling pathway by impairing Piezo1 currents and localization.

### Piezo1 and LecB interactions: one potential mechanism

When investigating potential interactions of EGFP-mPiezo1 with LecB-biotin by pull-down assays, we were able to detect EGFP-mPiezo1 signals in the extract of 3 h. We observed a double band at the expected height at approx. 300 kDa. This double band can be explained by various glycosylated states, as the upper band shows fully glycosylated EGFP-mPiezo1 and the lower one core-glycosylated EGFP-mPiezo1. Li and colleagues observed the same pattern when performing Western Blots against the human Piezo1 linked to EGFP with an anti-GFP antibody [64]. It is important to mention that EGFP-mPiezo1 signals were also observed in the pull-down supernatants of cells treated with LecB-biotin. This can be explained by the fact that there is also intracellular Piezo1, e. g. in mitochondrial membranes [74], which normally cannot interact with LecB that binds exclusively to the extracellular leaflet of the plasma membrane. Unfortunately, with this assay it is not possible to distinguish between direct and indirect interactions via other molecules, nor the type of interaction. However, when l-fucose was used to saturate the carbohydrate binding sites of LecB, thus preventing binding to glycosylated membrane receptors, no Piezo1 signal was detectable in the extracts. This led us to the conclusion that the binding of LecB to glycans is crucial for of interactions with Piezo1.

For the readout of the initial pull-down studies, an anti-Piezo1 antibody against human and murine Piezo1 was applied (Fig. S18). The use of this antibody indicated also an interaction of LecB with Piezo1 already after 30 min of LecB-biotin incubation, additionally to the 3-h time point. Considering the fluorescent images of EGFP-mPiezo1 and LecB-AF647 and the corresponding quantification of the MCC, an interaction at this early time point can be assumed. However, by optimizing the pull-down assay, using a highly specific GFP antibody, we could only confirm a clear interaction between EGFP-mPiezo1 and LecB-AF647. Overall, the GFP-antibody was later preferred due to poor availability of specific and reliable Piezo1 antibodies.

To investigate potential interaction possibilities between LecB and Piezo1, we performed computer simulations. Using modeling approaches, we were able to show that the cap region of Piezo1 and LecB exhibit a preferential mode of interaction due to the presence of electrostatic surface patches. In general, such surface patches, which are created by the distribution of the electrostatic potential, are important for molecular recognition, the formation of protein–protein complexes and the binding of antibodies to antigens, for example [75]. Considering our experimental evidence that the interactions between Piezo1 and LecB were uncoupled by blocking the carbohydrate binding sites of LecB by l-fucose, we speculate that the binding of LecB to the carbohydrate chains of Piezo1 may further enhance their interactions. Interactions between Piezo1 and LecB via amino acid contact, which mainly involve charged and polar residues, are seemingly less specific than the interactions of proteins with carbohydrates. However, in some cases it is well established that glycan-binding proteins can interact with non-carbohydrate elements of glycosylated proteins and lipid scaffolds of glycolipids in ways that strengthen both the affinity and binding [76]. On the other hand, these long-range electrostatic interactions could align LecB with respect to the cap of Piezo1 and thus increase the frequency of collisions and facilitate the recognition of a specific carbohydrate moiety of the glycan chain. Indeed, our simulations of protein–protein interactions showed that LecB formed a complex with the cap of Piezo1 around sequons. The sequons Asn-X-Ser/Thr (where X is any but not Pro residue) are N-glycosylated by a core glycan Manα1–3(Manα1–6)Manβ1-4GlcNAcβ1–4GlcNAcβ1–(Asn) which forms two arms of complex or hybrid glycans [64]. The lack of experimental data on the sequence of the N-glycans of the cap region prevented a more detailed analysis of intermolecular interactions. Nonetheless, the predicted mutual alignment of LecB and the cap of Piezo1 allowed us to infer a symmetry in the complex in which two fucose-binding sites of LecB are equidistant to two asparagine residues of the cap of Piezo1, which are N-glycosylated. This observation allowed us to develop a putative model for the interactions between LecB and Piezo1 via glycans. According to this model, the Lewis A (Le^a^) epitope, which was directly attached to the core N-glycan motive, could be stretched towards the binding site of LecB and occupy it, in the same way as already revealed by X-ray crystallography of the LecB-Le^a^ complex [65]. During the interaction with the cap of Piezo1, two symmetric N-glycans can be bound simultaneously by two carbohydrate-binding sites of LecB. We demonstrate the shortest possible carbohydrate chain length and show a simple way to realize the binding of LecB to Piezo1 in an efficient, multivalent manner, which increases specificity through avidity [88, 89]. Referring to the full Piezo1 construct expressed in red blood cells and lung tissue, Li et al. estimated the total mass of N-glycans to be 20–30 kDa [64]. Our calculations suggest that two neighboring short N-glycan chains of the cap, each of about 2 kDa (indicated for the core and one 1,6-arm of the N-glycan), are sufficient for multivalent LecB binding. It has been shown experimentally that glycan chains affected the stretch-activated currents through Piezo1 [64]. Under physiological conditions, this may be due to the interactions of N-glycans with other membrane proteins or the extracellular matrix, providing cells with an additional dynamic mechanism to regulate mechanosensitivity [64]. Glycans may facilitate interaction with binding partners such as PECAM-1 [43], E-cadherin [77] or specific glycolipids [78], which could alter the ability of the Piezo1 to sense membrane tension [79]. In the case of integrins, for example, N-glycans are important for clustering [64]. LecB may perturb and reorganize these interactions due to binding to Piezo1 or its neighbouring molecular partners.

## Conclusion

Our results show that the *Pseudomonas aeruginosa* lectin LecB interacts with the SAC Piezo1 channel changing its localization and the amplitude of the recorded stretch-induced currents. LecB effects on Piezo1 are time-dependent: after 30 min of incubation, LecB leads to higher stretch-induced Piezo1 currents in EGFP-mPiezo1-overexpressing cells with no significant changes in Piezo1 localization in the plasma membrane. After 3 h of LecB incubation, stretch-induced Piezo1 currents are lower compared to controls (in both EGFP-mPiezo1-overexpressing and wild type cells, with endogenous Piezo1) and EGFP-mPiezo1 is translocated to the posterior part of and the perinuclear area of the cell, potentially explaining lower currents. Our model suggests that LecB binds to the cap region of Piezo1, in which two carbohydrate-binding sites of LecB are occupied by two glycans derived from Piezo1. The other two opposite carbohydrate binding sites of the LecB tetramer can interact with glycans derived from the blades of Piezo1 and/or neighboring glycosylated proteins such as integrins and/or glycosphingolipids, causing conformational changes in the Piezo1 structure by pulling and pushing and affecting channel activity and modulating important signaling pathways related to cell migration, proliferation and wound healing.

## Supplementary Information

Below is the link to the electronic supplementary material.

Supplementary file1 (DOCX 8.20 MB)

## Data Availability

The datasets generated and/or analyzed during the current study are available from the corresponding author on reasonable request.

## Abbreviations

BSA: Bovine serum albumin
CAMs: Cell adhesion molecules
CDH5: Cadherin 5
DMEM: Dulbecco’s modified eagle’s medium
ECL: Enhanced chemiluminescence
EGFP: Enhanced green fluorescent protein
FCS: Fetal calf serum
Gb3: Globotriaosylceramide
HSPC: High-speed pressure clamp
IGF1R: Insulin-like growth factor receptor 1
LecA: *P. aeruginosa* Lectin A
LecB: *P. aeruginosa* Lectin B
MD: Molecular dynamics
MOI: Multiplicity of infection
PBS: Phosphate buffered saline
PDB: Protein data bank
PECAM-1: Platelet and endothelial cell adhesion molecule 1
*P. aeruginosa*: *Pseudomonas aeruginosa*
PFA: *para*-Formaldehyde
PEI: Polyethylenimine
PME: Particle mesh ewald
RPMI: Roswell park memorial institute
RT: Room temperature
SAC: Stretch-activated channel
TBS-T: Tris-buffered saline with 0.05% Tween-20
WT: Wild-type
