# Supplementary material for: LecB from Pseudomonas aeruginosa modulates Piezo1 currents and localization in a time-dependent manner
Source: Cell Mol Life Sci. 2025 Nov 14;82(1):399. doi: 10.1007/s00018-025-05934-z (PMC12618757; doi:10.1007/s00018-025-05934-z)
Supplement: Supplementary file 1 — Supplementary file1 (DOCX 8.20 MB) [file 18_2025_5934_MOESM1_ESM.docx]

**LecB from *Pseudomonas aeruginosa* modulates Piezo1 currents and localization in a time-dependent manner**

Anna-Sophia Kittel^1,2,3^, Olga N. Makshakova^1,2^, Michael Hauerwas^1,2^, Nikita Edel^1,2^, Niklas Knickmeier^1,2^, Jana Tomisch^1,2^, Ahmad Aljohmani^4^, Daniela Yildiz^4^, Rémi Peyronnet^,5,6*^ and Winfried Römer^1,2,*^

*^1^ Faculty of Biology, University of Freiburg, Freiburg, Germany*

*^2^ Signalling Research Centres BIOSS and CIBSS, University of Freiburg, Freiburg, Germany*

*^3^ Faculty of Chemistry and Pharmacy, University of Freiburg, Freiburg, Germany*

*^4^ Institute of Experimental and Clinical Pharmacology, PZMS, ZHMB, Saarland University, Homburg, Germany*

*^5^ Faculty of Medicine, University of Freiburg, Freiburg, Germany*

*^6^ Institute for Experimental Cardiovascular Medicine, University of Freiburg, Freiburg, Germany.*

^*^ Correspondence to

Winfried Römer ([winfried.roemer@bioss.uni-freiburg.de](mailto:winfried.roemer@bioss.uni-freiburg.de)), Schänzlestraße 18, University of Freiburg, 79104 Freiburg, Germany

and

Rémi Peyronnet ([remi.peyronnet@uniklinik-freiburg.de](mailto:remi.peyronnet@uniklinik-freiburg.de)), Elsässer Str. 2Q, University Clinic Freiburg, 79110 Freiburg, Germany


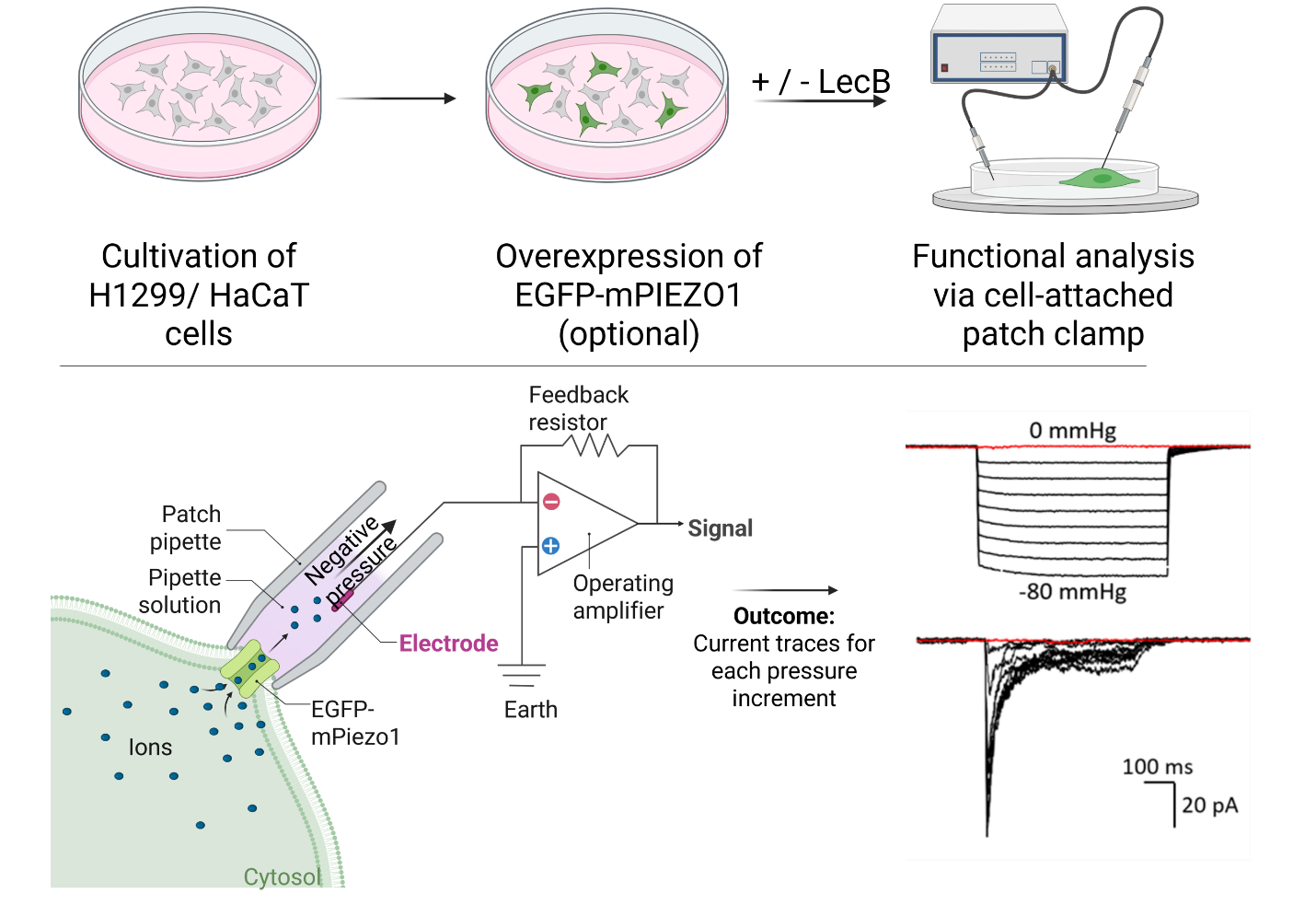


Figure S1: Electrophysiology workflow. H1299 lung epithelial cells either as wild-type and transiently transfected with N1-EGFP-mPiezo1 plasmid, and HaCaT cells were used for electrophysiological recordings. In the case of transfected cells, green fluorescently labeled cells were selected for cell-attached high-speed pressure clamp. Voltage was clamped at -80 mV and pressure pulses starting from 0 mmHg to -80 mmHg with increments of 10 mmHg were applied. Created with BioRender.


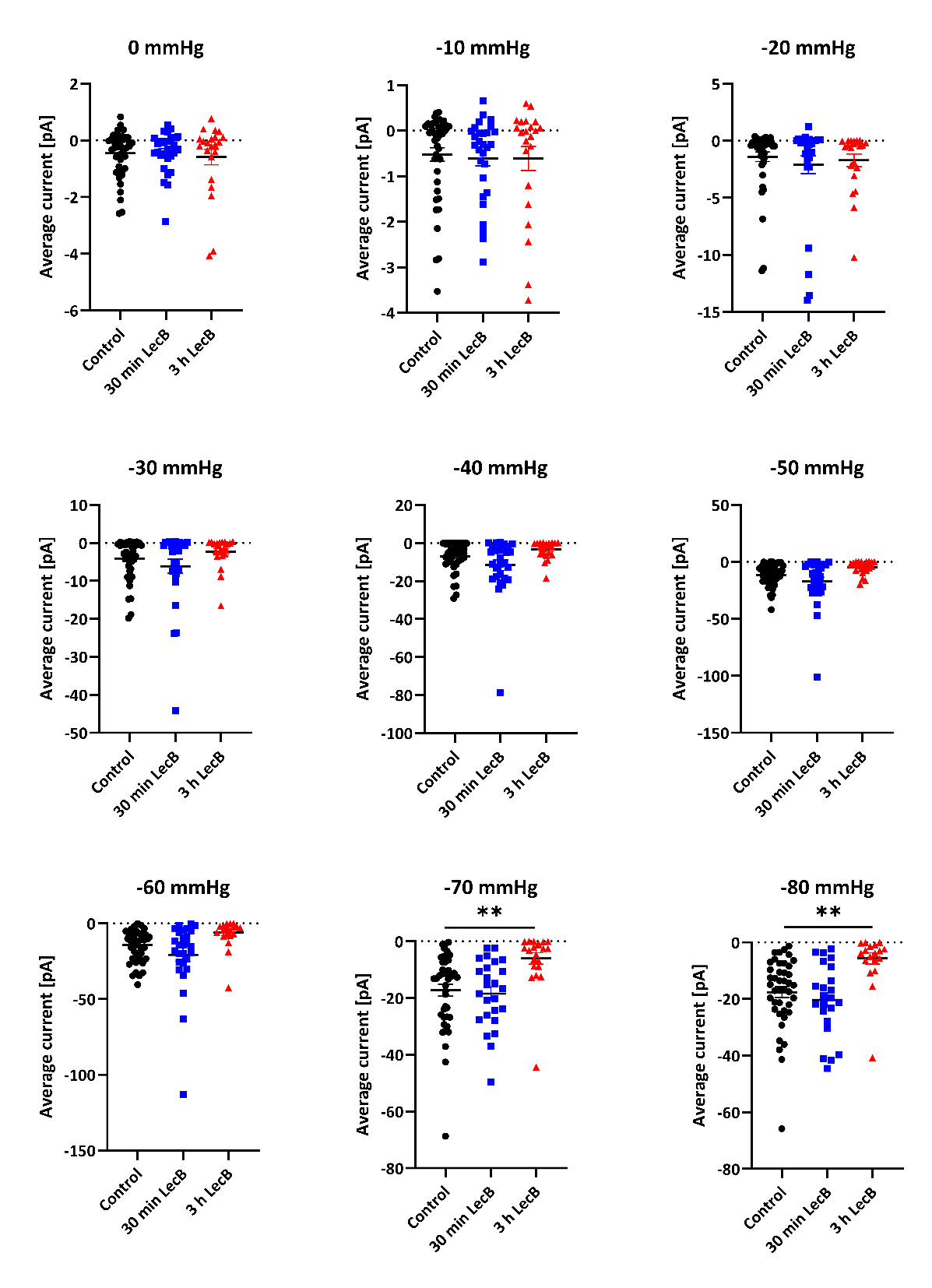


Figure S2: Average currents recorded in H1299 wild-type cells by cell-attached high speed pressure clamp after 30 minutes (blue) and 3 hours (red) of LecB treatment or untreated (control, black). Mean values are depicted as black bars. n _control_ = 42, n _LecB 30 min_ = 23-29, n _LecB 3 h_ = 21-22 patched cells. Variations in n are due to lost seals during recordings. Statistical significance was assessed with ANOVA and Dunnett’s multiple comparison test. * p ≤ 0.05; ** p ≤ 0.01; *** p ≤ 0.001; **** p ≤ 0.0001; not statistically relevant values are not depicted.


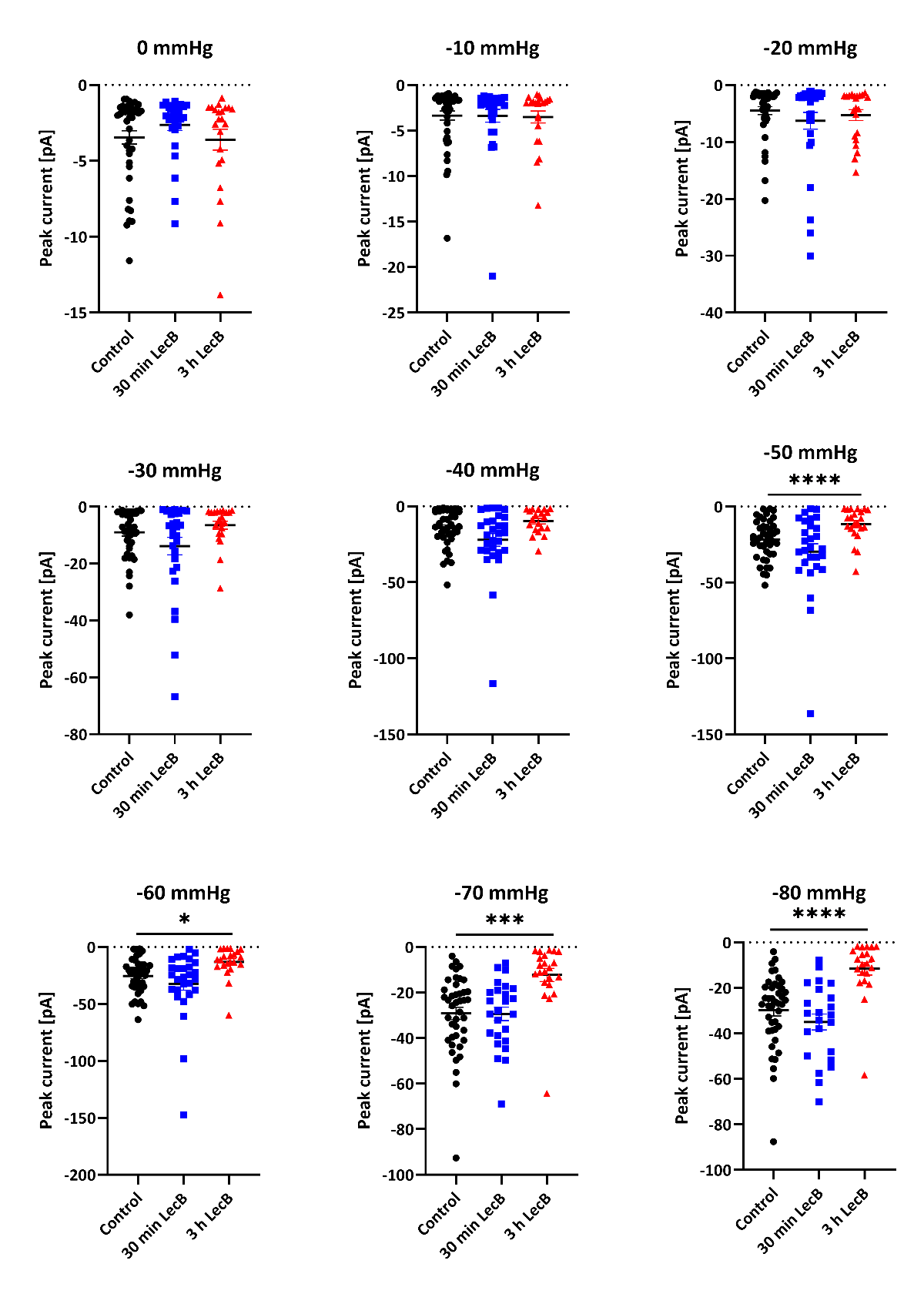


Figure S3: Peak currents recorded in H1299 wild-type cells by cell-attached high speed pressure clamp after 30 minutes (blue) and 3 hours (red) of LecB treatment or untreated (control, black). Mean values are depicted as black bars. n _control_ = 42, n _LecB 30 min_ = 23-29, n _LecB 3 h hh_= 21-22 patched cells. Variations in n are due to lost seals during recordings. Statistical significance was assessed with ANOVA and Dunnett’s multiple comparison test. * p ≤ 0.05; ** p ≤ 0.01; *** p ≤ 0.001; **** p ≤ 0.0001; not statistically relevant values are not depicted.


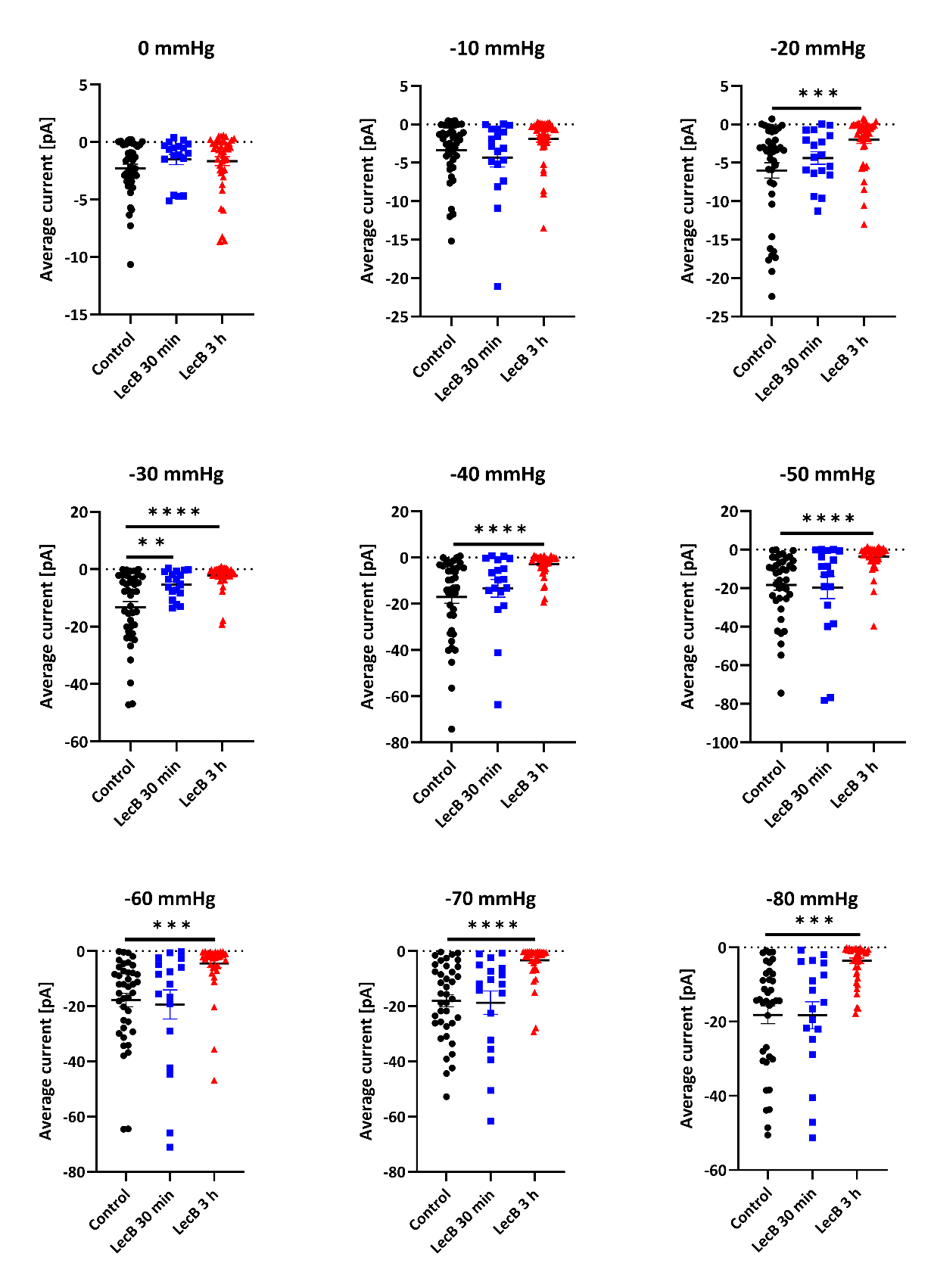


Figure S4: Average currents recorded in HaCaT cells by cell-attached high speed pressure clamp after 30 minutes (blue) and 3 hours (red) of LecB treatment or untreated (control, black). Mean values are depicted as black bars. n _control_ = 37-42, n _LecB 30 min_ = 18, n _LecB 3 h_ = 42 patched cells. Variations in n are due to lost seals during recordings. Statistical significance was assessed with ANOVA and Dunnett’s multiple comparison test. * p ≤ 0.05; ** p ≤ 0.01; *** p ≤ 0.001; **** p ≤ 0.0001; not statistically relevant values are not depicted.


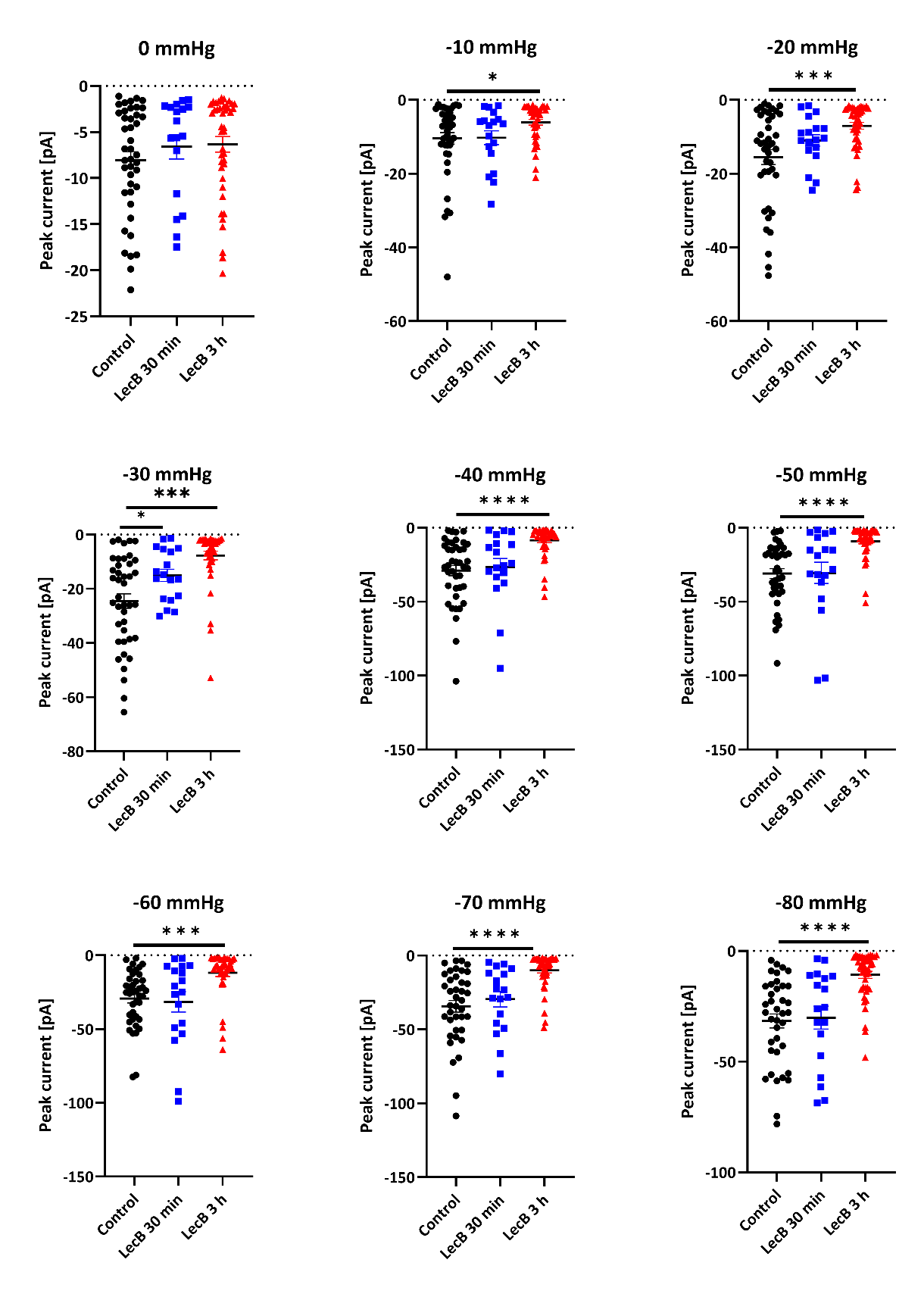


Figure S5: Peak currents recorded in HaCaT cells by cell-attached high speed pressure clamp after 30 minutes (blue) and 3 hours (red) of LecB treatment or untreated (control, black). Mean values are depicted as black bars. n _control_ = 37-42, n _LecB 30 min_ = 18, n _LecB 3 h_ = 42 patched cells. Variations in n are due to lost seals during recordings. Statistical significance was assessed with ANOVA and Dunnett’s multiple comparison test. * p ≤ 0.05; ** p ≤ 0.01; *** p ≤ 0.001; **** p ≤ 0.0001; not statistically relevant values are not depicted.


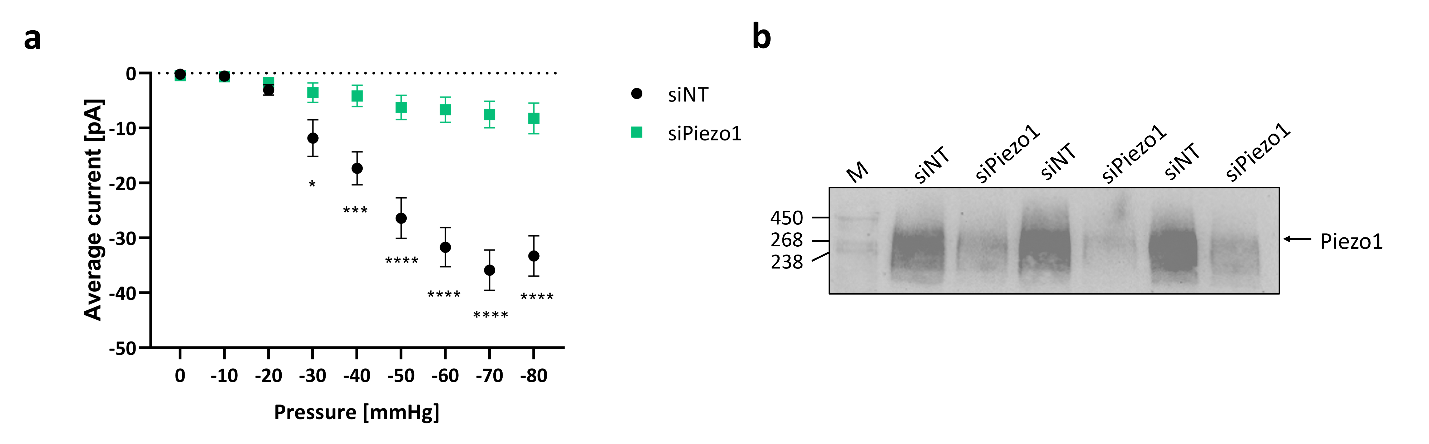


Figure S6: **(a)** Average currents recorded in H1299 cells by cell-attached high speed pressure clamp after treatment with siRNA against Piezo1 (siPiezo1) for 72 hours. Cells that were treated with non-targeting siRNA (siNT) served as control. Mean values are depicted as bars. n _siPiezo1_ = 19-20, n _siNT_ = 32 patched cells. Variations in n are due to lost seals during recordings. Statistical significance was assessed with t-test. * p ≤ 0.05; ** p ≤ 0.01; *** p ≤ 0.001; **** p ≤ 0.0001; not statistically relevant values are not depicted. **(b)** Western Blot of H1299 cells that were treated with either siNT or siPiezo1 siRNA for 72 hours against Piezo1. Cell lysates were obtained from three biological replicates.


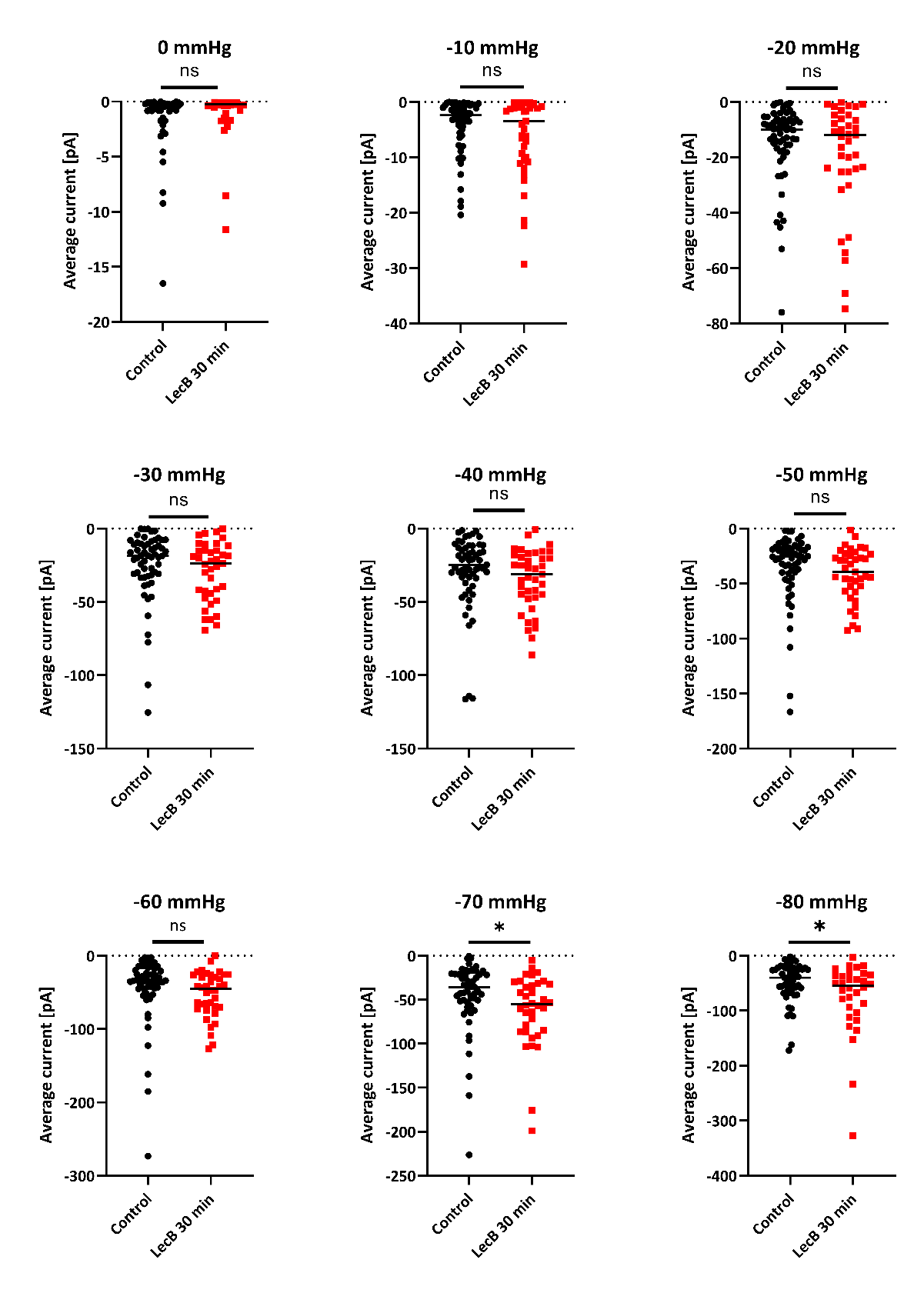


Figure S7: Average currents recorded in EGFP-mPiezo1-expressing H1299 cells by cell-attached high speed pressure clamp after 30 minutes of LecB treatment (red) or untreated (control, black). Mean values are depicted as black bars. n _control_ = 53-59, n _LecB 30 min_ = 32-39 patched cells. Variations in n are due to lost seals during recordings. Significance was assessed with an unpaired t-test, assuming Gaussian distribution. * p ≤ 0.05; ns: not statistically relevant.


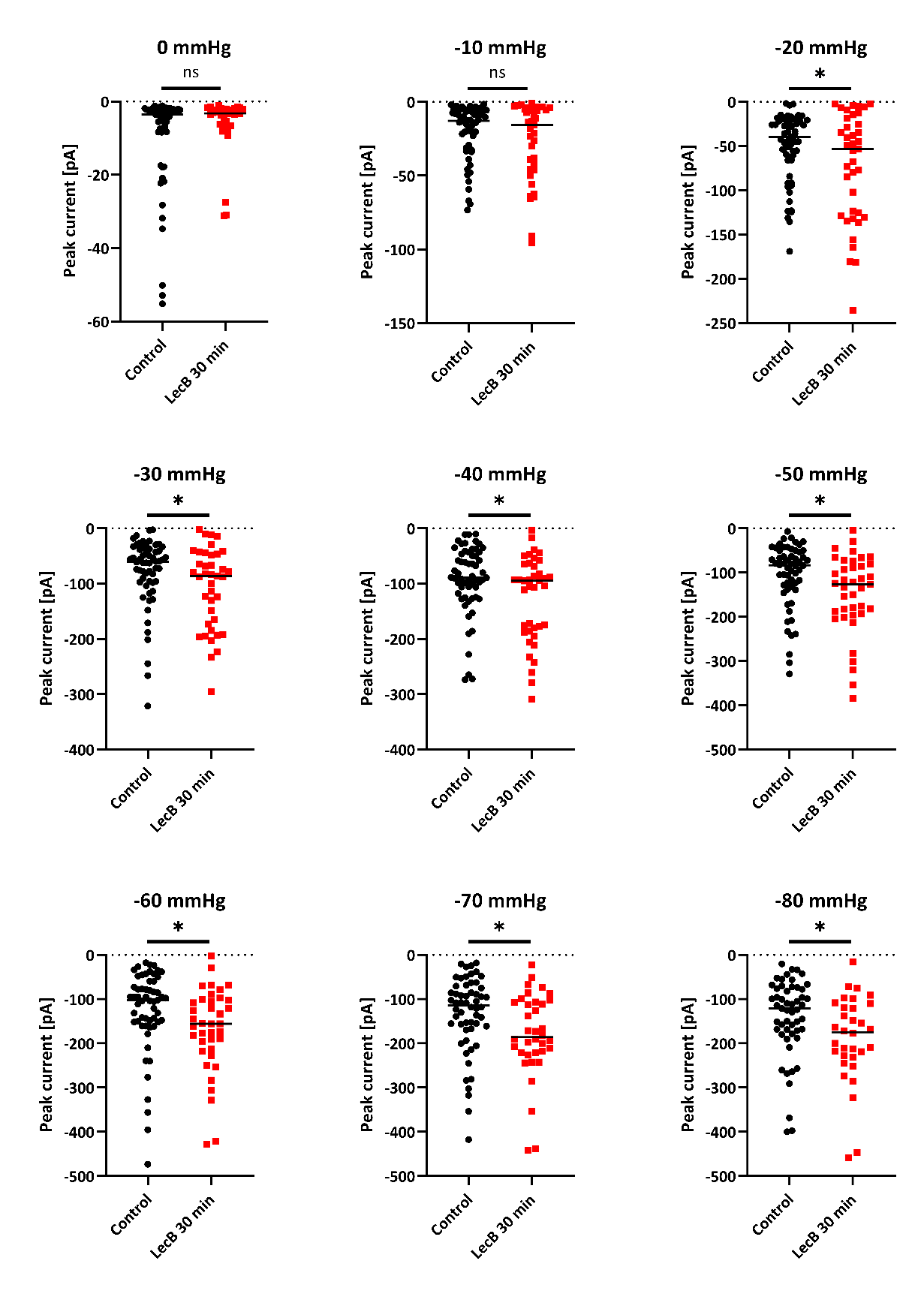


Figure S8: Peak currents recorded in EGFP-mPiezo1-expressing H1299 cells by cell-attached high speed pressure clamp after 30 minutes of LecB treatment (red) or untreated (control, black). Mean values are depicted as black bars. n _control_ = 53-59, n _LecB 30 min_ = 32-39 patched cells. Variations in n are due to lost seals during recordings. Significance was assessed with an unpaired t-test, assuming Gaussian distribution. * p ≤ 0.05; ** p ≤ 0.01; *** p ≤ 0.001; **** p ≤ 0.0001; ns: not statistically relevant.


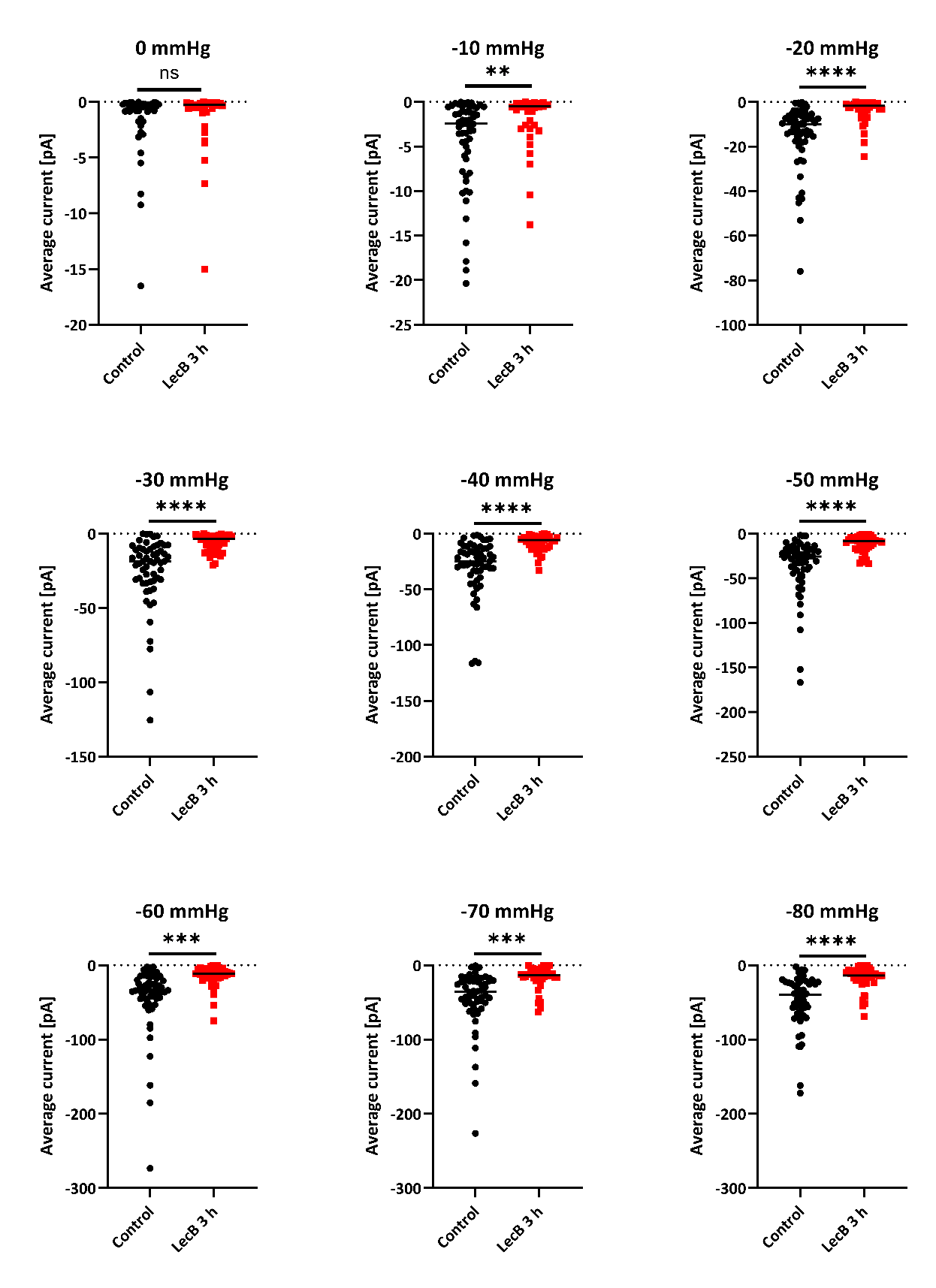


Figure S9: Average currents recorded in EGFP-mPiezo1-expressing H1299 cells by cell-attached high speed pressure clamp after 3 hours of LecB treatment (red) or untreated (control, black). Mean values are depicted as black bars. n _control_ = 53-59, n _LecB 3 h_ = 42 patched cells. Variations in n are due to lost seals during recordings. Significance was assessed with an unpaired t-test, assuming Gaussian distribution. * p ≤ 0.05; ** p ≤ 0.01; *** p ≤ 0.001; **** p ≤ 0.0001; ns: not statistically relevant.


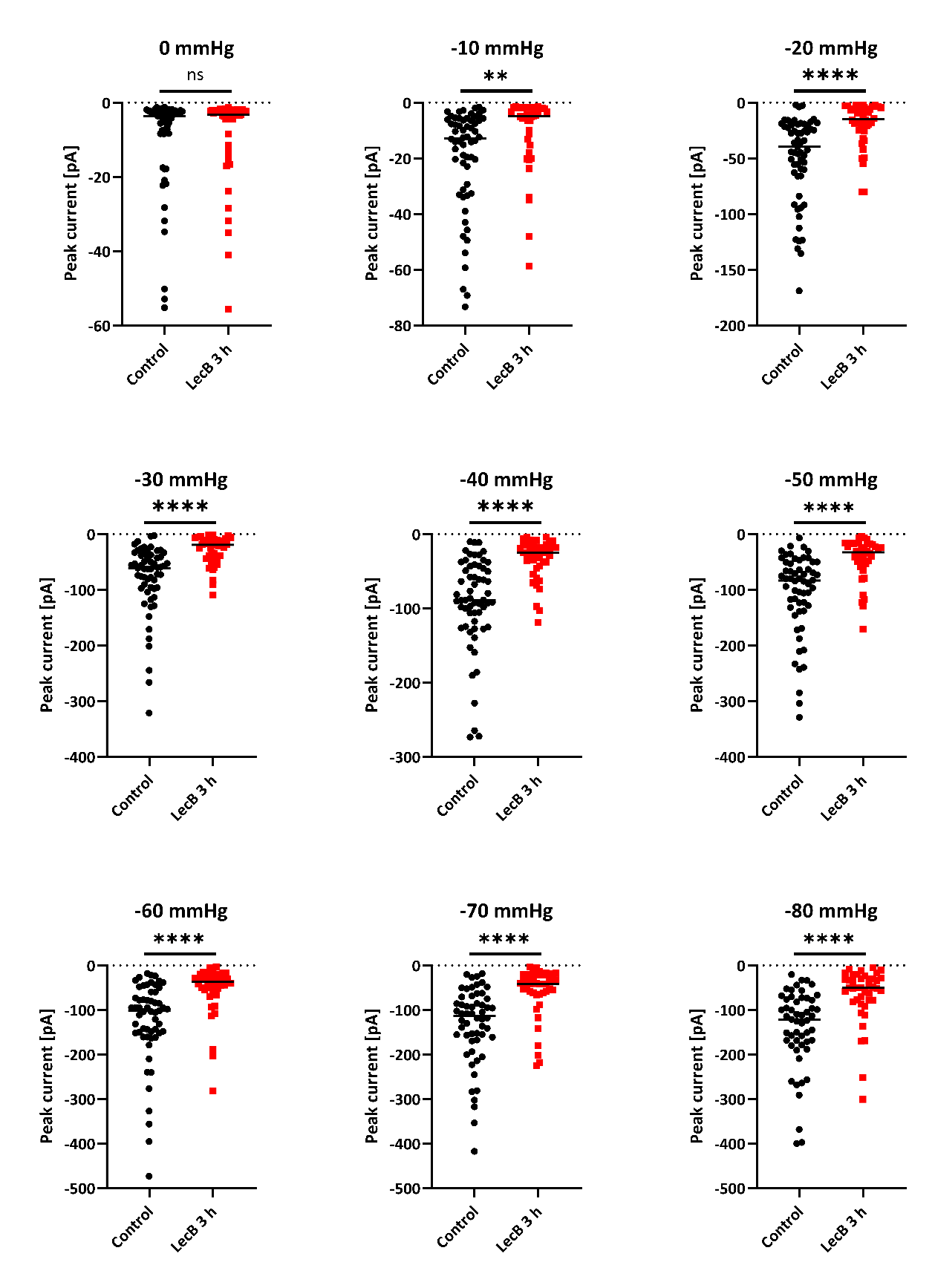


Figure S10: Peak currents recorded in EGFP-mPiezo1-expressing H1299 cells by cell-attached high speed pressure clamp after 3 hours of LecB treatment (red) or untreated (control, black). Mean values are depicted as black bars. n _control_ = 53-59, n _LecB 3 h_ = 42 patched cells. Variations in n are due to lost seals during recordings. Significance was assessed with an unpaired t-test, assuming Gaussian distribution. * p ≤ 0.05; ** p ≤ 0.01; *** p ≤ 0.001; **** p ≤ 0.0001; ns: not statistically relevant.


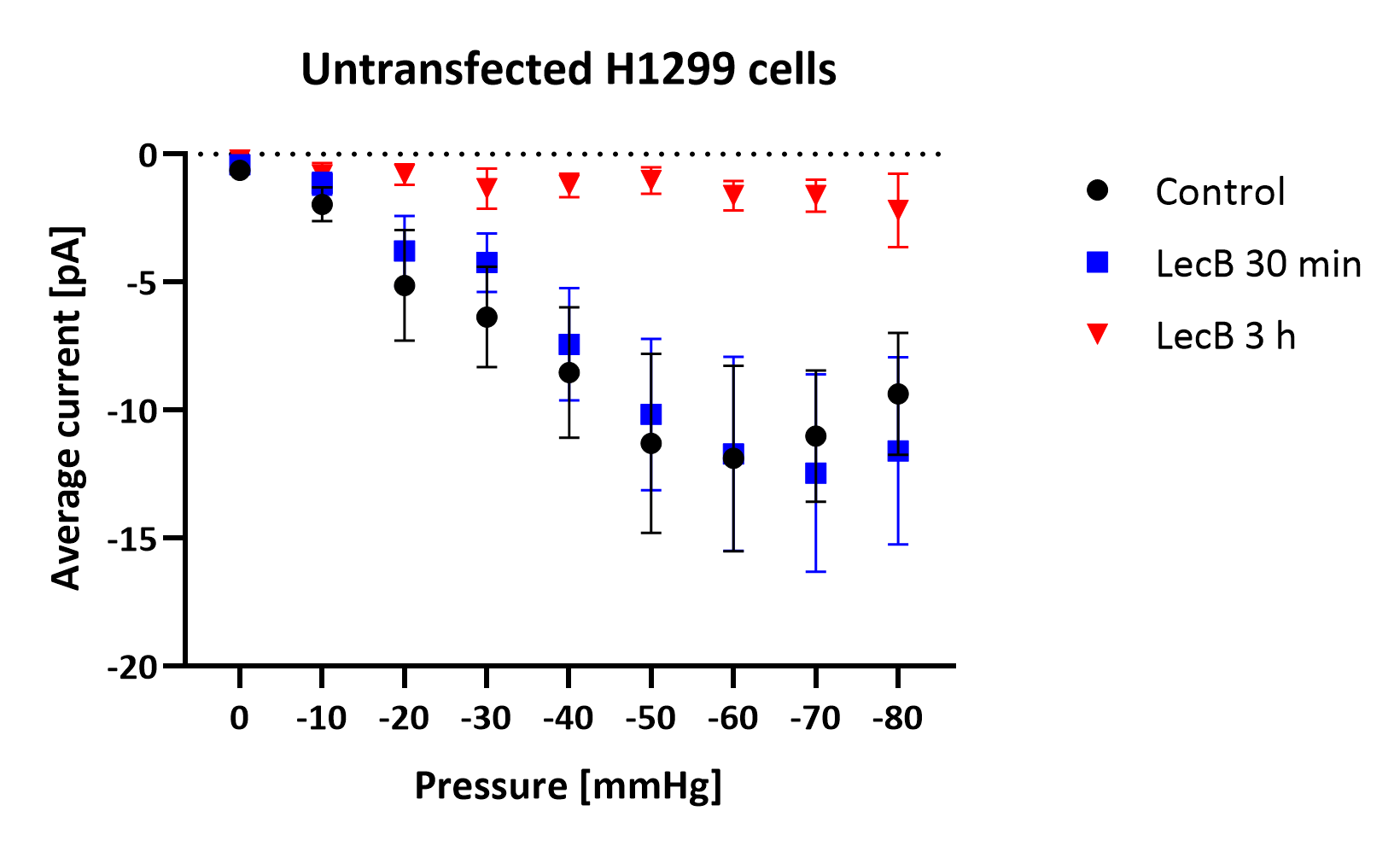


Figure S11: Average currents recorded in EGFP-expressing H1299 cells by cell-attached high speed pressure clamp after 30 minutes (red, n = 9) and 3 hours (blue, n = 10) of LecB treatment or untreated (control, black, n = 15). SEM values are depicted as black bars.


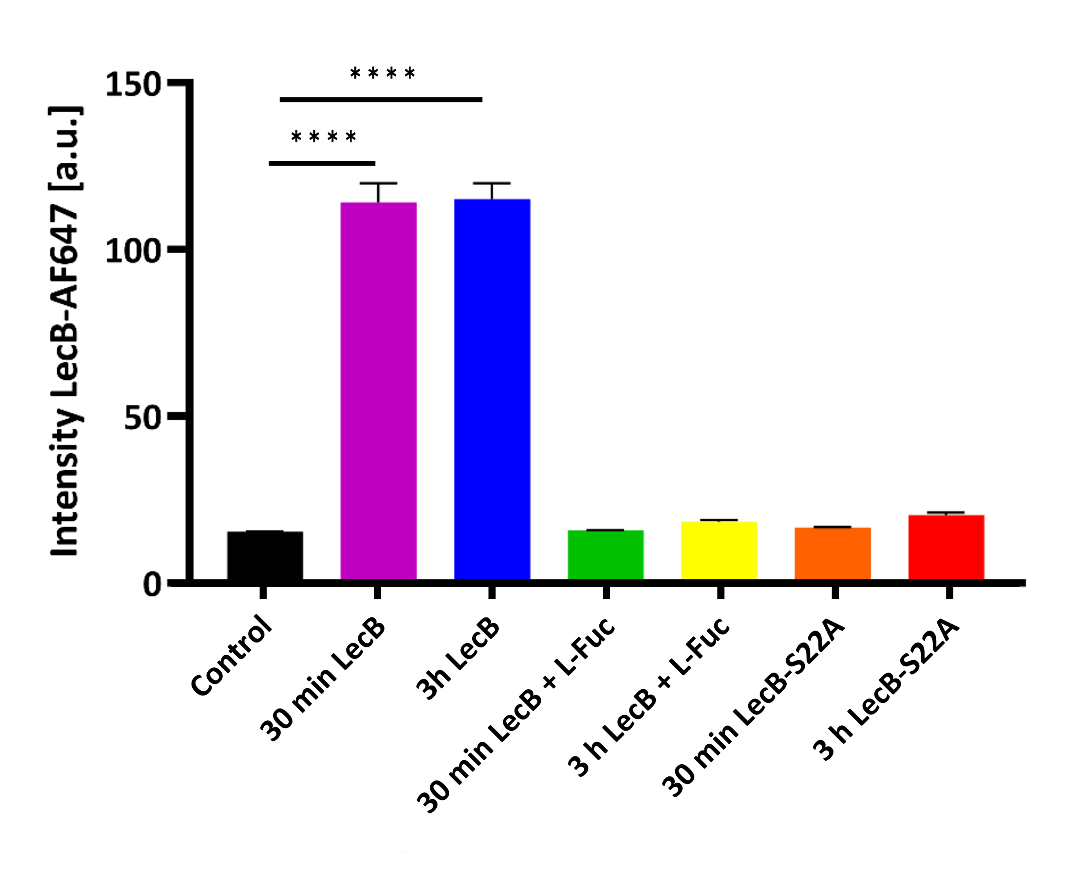


Figure S12: Fluorescence intensities of the LecB-AF647 signal in EGFP-mPiezo1-expressing H1299 cells. Cells were treated for 30 minutes with LecB-AF647 (n = 66), for 3 hours with LecB-AF647 (n =104), for 30 minutes with LecB-AF647 and L-Fucose (L-Fuc, n = 44), for 3 hours with LecB-AF647 and L-Fuc (n = 36), or for 30 minutes (n = 44) and 3 hours (n = 27) with the carbohydrate-binding site mutant LecB-S22A-AF647. Untreated cells served as control (n = 87). Images were acquired by confocal microscopy and intensities were obtained via FIJI. A one-way ANOVA, with multiple comparison test was performed. **** = p ≤ 0.0001. Only significant values are depicted.


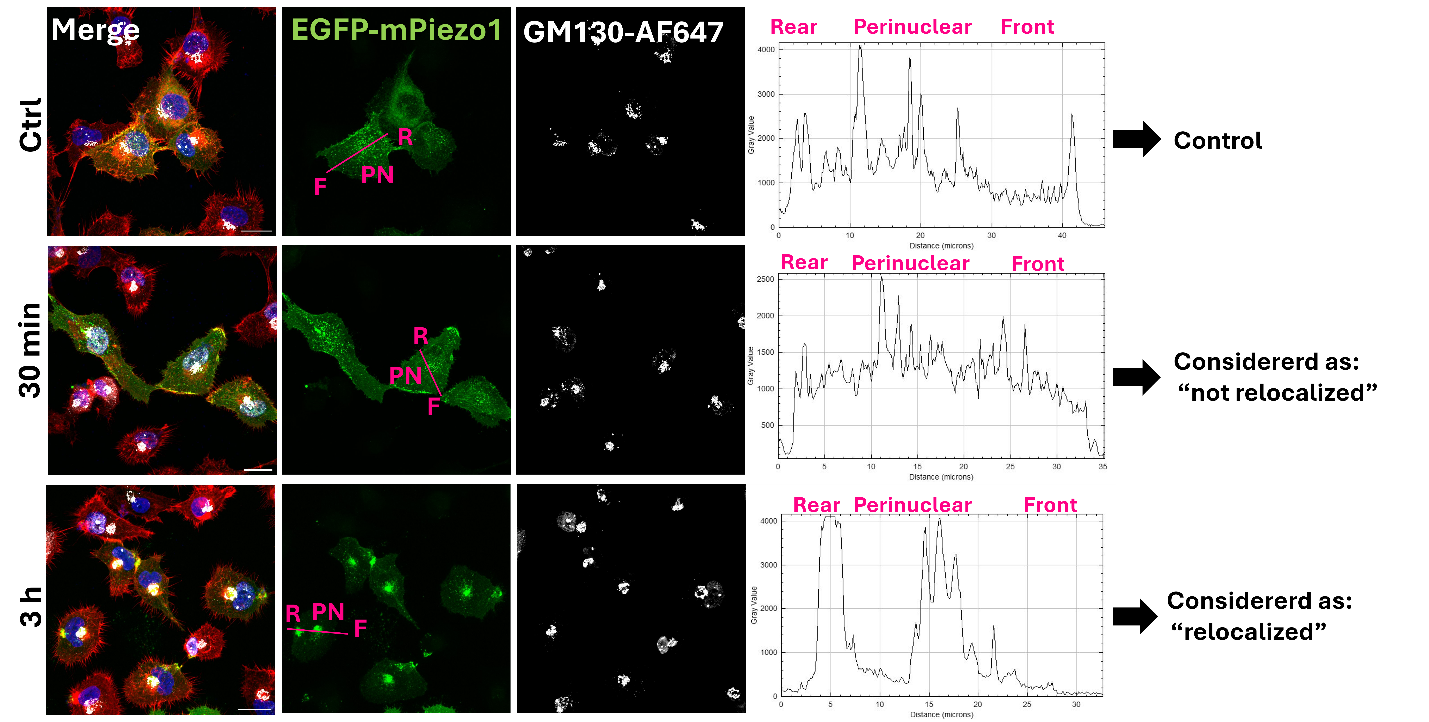
Figure S13: Schematic diagram illustrating the measurement of fluorescence intensity along the front-rear axis of EGFP-mPiezo1-expressing H1299 cells. A line was drawn along the front-rear axis (regarding the position of the Golgi apparatus and the nucleus) of EGFP-mPiezo1-expressing H1299 cells. R = rear of the cell,
PN = perinuclear area, F = front of a cell. Intensity profiles of EGFP-mPiezo1 were analyzed and an intensity shift towards the cell rear and the perinuclear area was considered as “relocalization” of EGFP-mPiezo1. Scale bars: 20 µm.


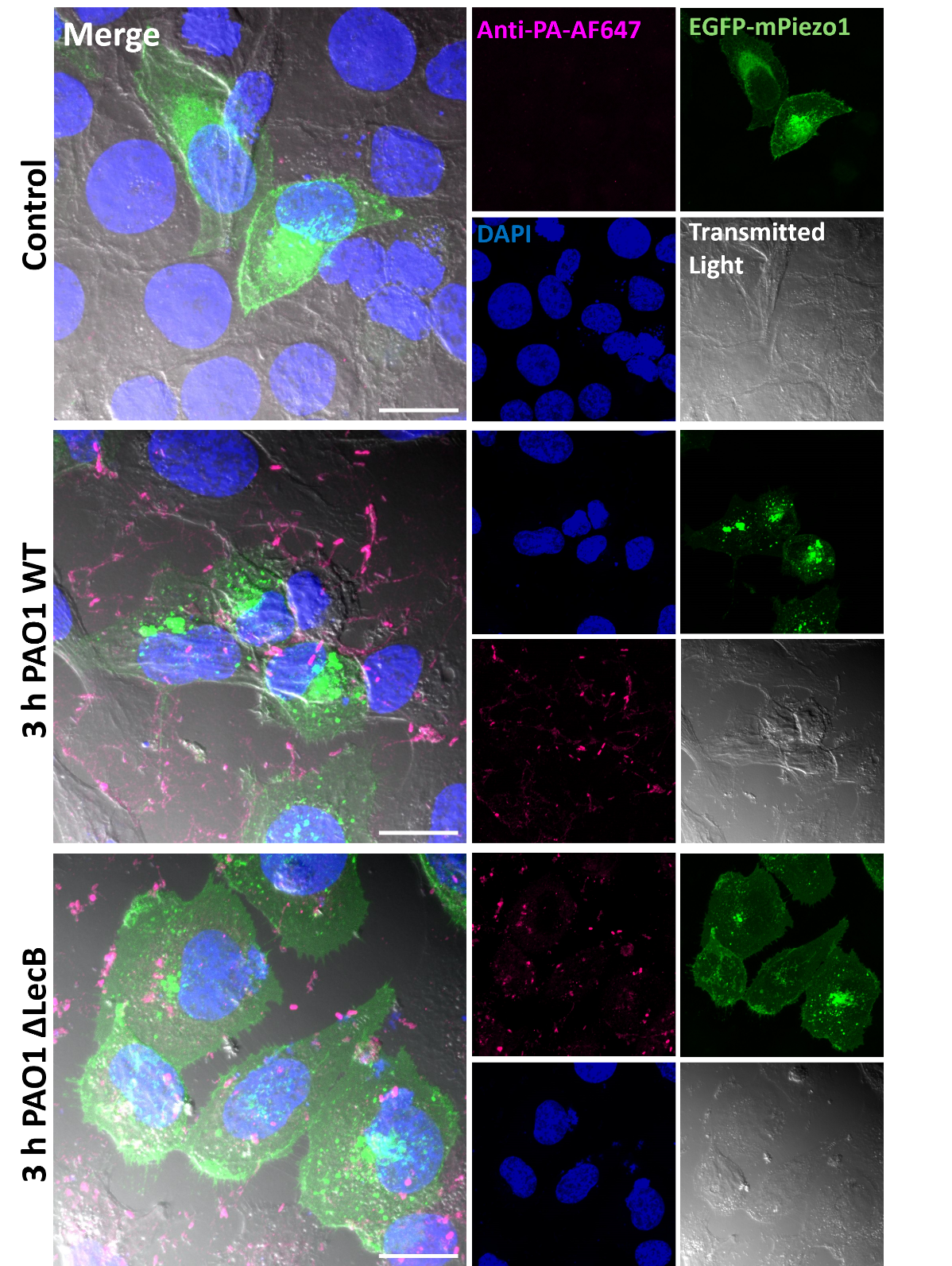


Figure S14: The bacterium Pseudomonas aeruginosa changes the intracellular distribution of EGFP-mPiezo1. EGFP-mPiezo1-expressing H1299 cells were incubated with the Pseudomonas aeruginosa PAO1 wild-type (WT) or ΔLecB strains (MOI 10) for 3 hours at 37 °C. Cells that were not treated with bacteria served as control. After treatment, the cells were washed with PBS and were fixed with 16 % PFA. For bacterial staining, cells were permeabilized with a PBS solution containing 0.2 % Saponin and 3 % BSA and then treated with anti-Pseudomonas aeruginosa (PA) antibody and subsequently with the respective secondary antibody linked with AF647, and DAPI, respectively. The presented images were acquired by confocal microscopy and Z projections are shown. Scale bars: 20 µm.


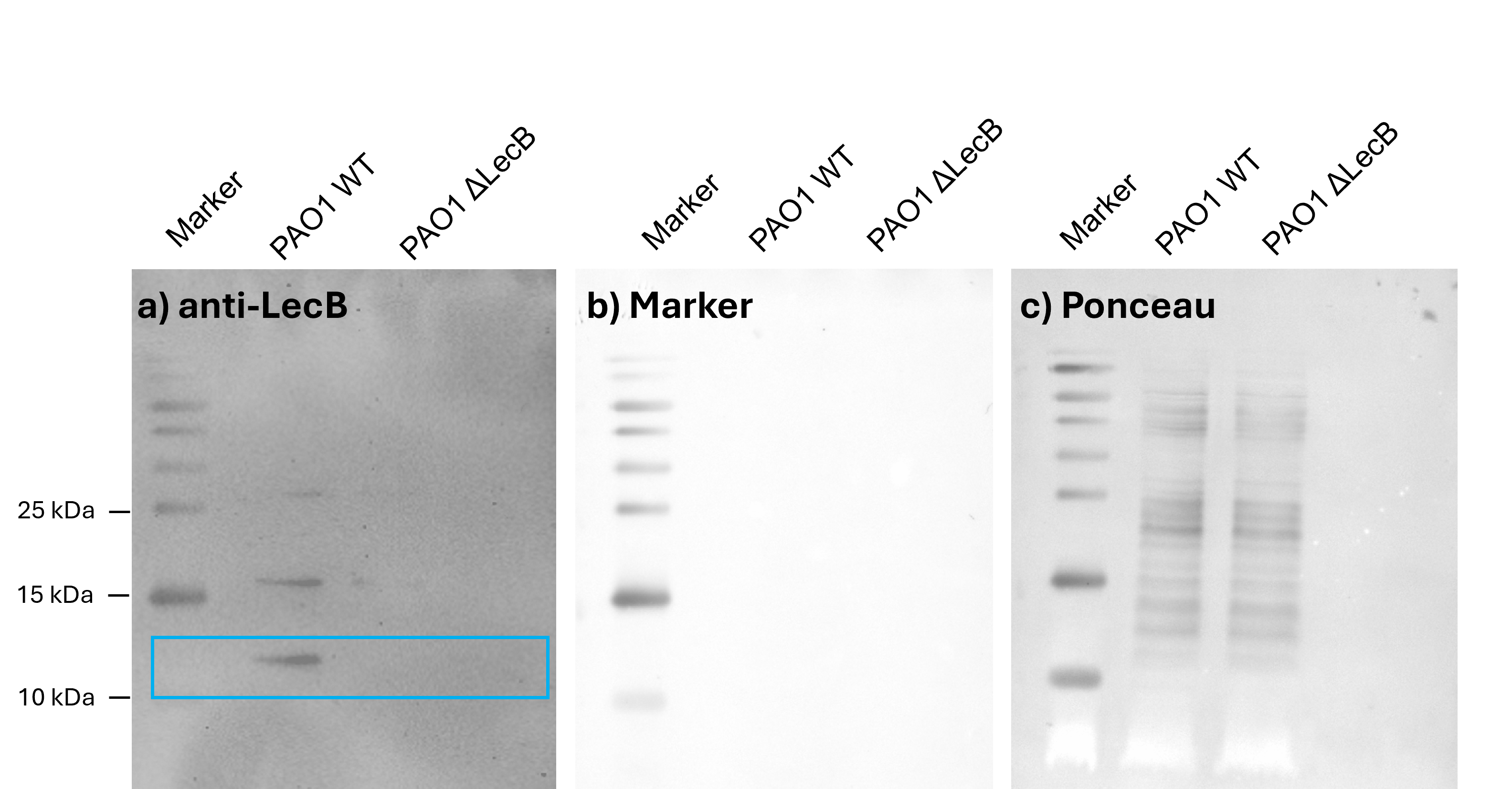


Figure S15: Western Blot of the P. aeruginosa PAO1 wild-type (PAO1 WT) and P. aeruginosa ΔLecB (PAO1 ΔLecB) strains confirms absence of LecB in the P. aeruginosa ΔLecB strain. Bacterial cultures were diluted with SDS running buffer and boiled for 10 min at 95 °C. Subsequent SDS PAGE and Western Blot with anti-LecB was performed, displayed in image a). A colorimetric blot of the membrane was obtained to show the marker (b) and a Ponceau staining was performed (c).


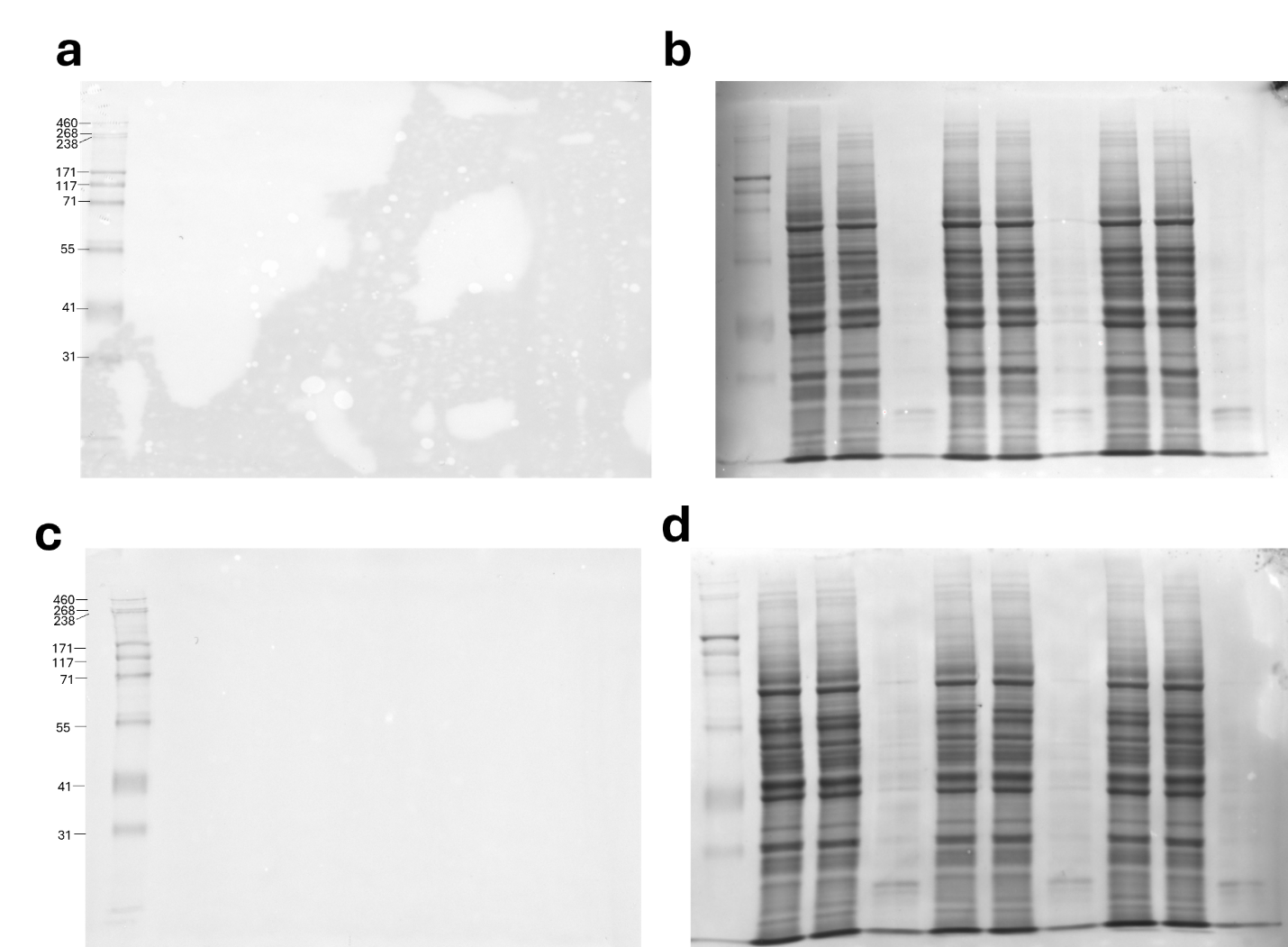


Figure S16: Marker and Ponceau staining of the pull-down with LecB-biotin. **(a)** Marker and **(b)** Ponceau staining for EGFP-mPiezo1-expressing H1299 cells that were treated with LecB-biotin for 30 min. **(c)** Marker and **(d)** Ponceau staining for EGFP-mPiezo1-expressing H1299 cells that were treated with LecB for 3 hours.


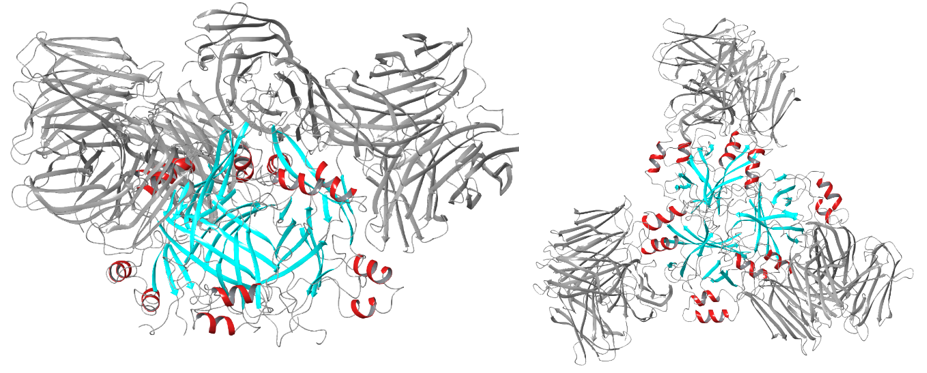


Figure S17: Side and top view of the complex of the cap trimer of Piezo1 and three LecB tetramers. Both proteins are displayed in cartoon presentation, the β-sheets of Piezo1 are shown in cyan and the α-helices in red, the LecB tetramer is represented in grey. The complex of the cap region of Piezo1 with LecB was equilibrated in the course of MD trajectories.


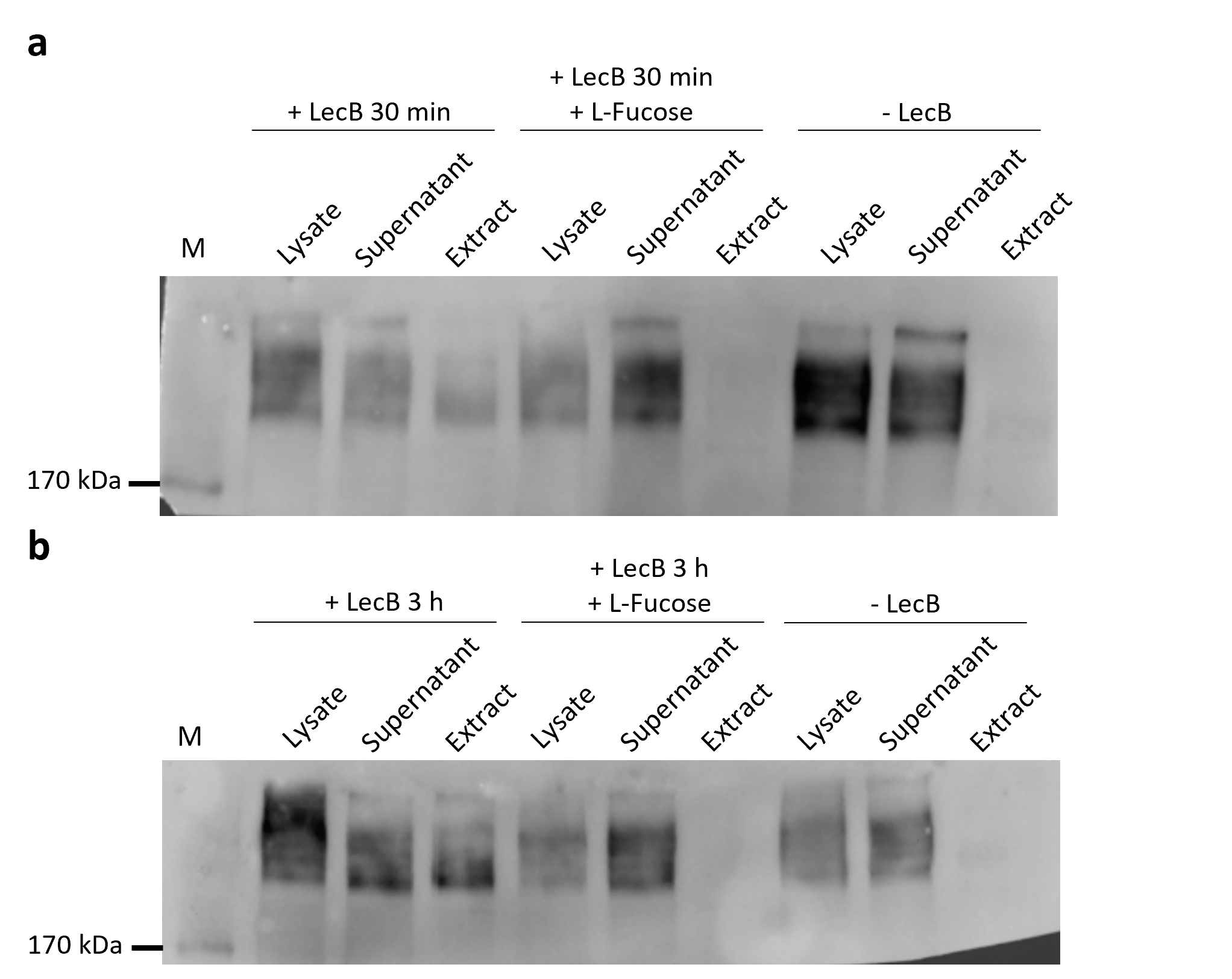


Figure S18: EGFP-mPiezo1-expressing H1299 cells were treated with LecB-biotin (1 μM) for 30 minutes and 3 hours, respectively, or remained untreated. To block LecB binding to glycosylated receptors at the plasma membrane, L-fucose (45 mM) was partly applied. After lysis, cell lysates were incubated with magnetic streptavidin beads. Lysate, supernatant and extract were run over a 6 % acrylamide SDS PAGE. PVDF membranes were stained with an anti-Piezo1 primary antibody followed by an anti-mouse horseradish peroxidase secondary antibody. M = Marker. **a** and **b**: Immunoblots depict Piezo1 signals of different strength respective to the applied treatment for 30 minutes (**a**) and 3 hours (**b**).

Table 1: Per-residue contacts between subunits of cap region of Piezo1 and LecB in the course of trajectories.

| CAP | | | LecB | | |
| --- | --- | --- | --- | --- | --- |
| Residue* | Mean distance, nm | Standard deviation, nm | Residue** | Mean distance, nm | Standard deviation, nm |
| Trajectory 1 |  |  | Subunit A |  |  |
| THR2349 | 0.18 | 0.0094 | ASP75 | 0.179 | 0.0101 |
| SER2365 | 0.202 | 0.0221 | ARG72 | 0.187 | 0.0259 |
| GLN2435 | 0.203 | 0.0191 | ASN34 | 0.198 | 0.0166 |
| TYR2309 | 0.204 | 0.0383 | LEU76 | 0.203 | 0.0163 |
| GLU2343 | 0.204 | 0.0691 | GLN64 | 0.206 | 0.0238 |
| ARG2402 | 0.206 | 0.023 | THR36 | 0.206 | 0.0214 |
| ASP2363 | 0.212 | 0.0289 | LYS62 | 0.219 | 0.0332 |
| PRO2369 | 0.227 | 0.0165 | GLN66 | 0.229 | 0.0198 |
| VAL2367 | 0.228 | 0.0187 | PRO73 | 0.229 | 0.0178 |
| GLN2356 | 0.23 | 0.0312 | LEU31 | 0.23 | 0.0181 |
| GLU2395 | 0.233 | 0.0303 | THR2 | 0.231 | 0.031 |
| GLN2364 | 0.235 | 0.0259 | GLY71 | 0.24 | 0.0202 |
| PRO2506 | 0.245 | 0.0815 | GLU35 | 0.241 | 0.0533 |
| GLN2404 | 0.254 | 0.0549 | VAL5 | 0.243 | 0.0348 |
| GLN2353 | 0.262 | 0.0392 | ALA1 | 0.261 | 0.0362 |
| ALA2350 | 0.264 | 0.0315 | SER68 | 0.262 | 0.0249 |
| ASP2505 | 0.267 | 0.1174 | GLN3 | 0.273 | 0.0344 |
| HIS2370 | 0.269 | 0.0254 | ASN29 | 0.287 | 0.0681 |
| SER2348 | 0.27 | 0.0295 | ASN70 | 0.295 | 0.0411 |
| PRO2362 | 0.286 | 0.0948 | SER74 | 0.359 | 0.0353 |
| ARG2352 | 0.294 | 0.065 |  |  |  |
| LEU2342 | 0.295 | 0.0802 |  |  |  |
| GLU2359 | 0.295 | 0.0754 | Subunit C |  |  |
| ARG2361 | 0.304 | 0.0924 | ASP75 | 0.213 | 0.0663 |
| PRO2508 | 0.317 | 0.1623 | TYR102 | 0.258 | 0.0548 |
| VAL2366 | 0.35 | 0.0538 | ASN100 | 0.26 | 0.0562 |
| ILE2368 | 0.353 | 0.0528 | ASN11 | 0.269 | 0.0875 |
| LEU2344 | 0.367 | 0.086 | ASN56 | 0.347 | 0.229 |
| GLU2433 | 0.378 | 0.0619 | GLY97 | 0.382 | 0.1136 |
| LYS2305 | 0.385 | 0.0797 | ARG13 | 0.385 | 0.3011 |
|  |  |  |  |  |  |
| Trajectory 2 |  |  |  |  |  |
| Subunit 1 |  |  | Subunit A |  |  |
|  |  |  |  |  |  |
| THR2349 | 0.18 | 0.0094 | ASP75 | 0.18 | 0.0094 |
| GLN2435 | 0.192 | 0.0205 | THR36 | 0.189 | 0.0162 |
| ASP2363 | 0.192 | 0.0244 | GLN66 | 0.191 | 0.0116 |
| GLN2364 | 0.193 | 0.0177 | LYS62 | 0.192 | 0.0244 |
| ARG2352 | 0.193 | 0.0341 | GLN64 | 0.193 | 0.0156 |
| GLN2353 | 0.194 | 0.012 | ASN34 | 0.194 | 0.0176 |
| PRO2362 | 0.197 | 0.0207 | ARG72 | 0.208 | 0.0496 |
| ARG2402 | 0.198 | 0.0198 | LEU76 | 0.211 | 0.0202 |
| ARG2361 | 0.208 | 0.0381 | THR2 | 0.214 | 0.0216 |
| VAL2367 | 0.21 | 0.0193 | ALA1 | 0.214 | 0.0188 |
| GLN2356 | 0.214 | 0.0188 | PRO73 | 0.226 | 0.016 |
| SER2365 | 0.217 | 0.0235 | GLU35 | 0.228 | 0.051 |
| PRO2369 | 0.225 | 0.0171 | LEU31 | 0.244 | 0.0209 |
| CYS2437 | 0.23 | 0.0218 | SER68 | 0.245 | 0.0225 |
| VAL2366 | 0.259 | 0.0213 | GLY71 | 0.253 | 0.0227 |
| TYR2309 | 0.269 | 0.0433 | ASN29 | 0.256 | 0.0412 |
| GLU2343 | 0.27 | 0.1379 | GLN3 | 0.26 | 0.0298 |
| GLN2404 | 0.272 | 0.0553 | VAL5 | 0.309 | 0.0416 |
| HIS2370 | 0.272 | 0.0344 | THR39 | 0.31 | 0.0679 |
| SER2348 | 0.277 | 0.0327 | ALA38 | 0.347 | 0.0562 |
| ALA2350 | 0.283 | 0.0318 | THR27 | 0.361 | 0.0862 |
| ASP2436 | 0.288 | 0.0322 | ALA37 | 0.362 | 0.0511 |
| LEU2342 | 0.294 | 0.09 | ASN70 | 0.383 | 0.1102 |
| ILE2368 | 0.307 | 0.0413 | SER74 | 0.393 | 0.0327 |
| GLU2433 | 0.337 | 0.032 |  |  |  |
| GLU2395 | 0.337 | 0.1119 | Subunit C |  |  |
| LEU2344 | 0.343 | 0.0622 | ASP75 | 0.189 | 0.0264 |
| GLU2359 | 0.345 | 0.0968 | ASN100 | 0.271 | 0.0491 |
| LYS2305 | 0.381 | 0.0895 | GLY97 | 0.391 | 0.0935 |
| ASN2310 | 0.399 | 0.0977 |  |  |  |
|  |  |  | Subunit A |  |  |
|  |  |  | GLU86 | 0.221 | 0.0705 |
|  |  |  |  |  |  |
| Subunit 2 |  |  | Subunit A |  |  |
|  |  |  |  |  |  |
| ALA2350 | 0.179 | 0.01 | ASP75 | 0.178 | 0.0101 |
| ILE2403 | 0.18 | 0.0083 | ARG72 | 0.179 | 0.009 |
| LEU2344 | 0.183 | 0.0139 | GLU35 | 0.18 | 0.0083 |
| ASP2436 | 0.191 | 0.0175 | ASN34 | 0.193 | 0.0203 |
| GLN2353 | 0.192 | 0.0323 | THR36 | 0.201 | 0.031 |
| GLU2396 | 0.199 | 0.0255 | LEU76 | 0.202 | 0.0155 |
| LEU2354 | 0.205 | 0.0184 | THR2 | 0.212 | 0.0193 |
| LEU2357 | 0.214 | 0.0338 | ALA1 | 0.218 | 0.0397 |
| LEU2371 | 0.231 | 0.0352 | GLY71 | 0.221 | 0.025 |
| ASP2397 | 0.234 | 0.045 | SER68 | 0.231 | 0.034 |
| ILE2368 | 0.241 | 0.0397 | GLN66 | 0.239 | 0.0463 |
| HIS2370 | 0.244 | 0.0272 | LEU31 | 0.242 | 0.0207 |
| ASN2310 | 0.25 | 0.0924 | GLN64 | 0.245 | 0.0383 |
| GLN2364 | 0.25 | 0.082 | PRO73 | 0.245 | 0.0197 |
| GLU2343 | 0.261 | 0.0235 | GLN3 | 0.269 | 0.036 |
| VAL2366 | 0.264 | 0.0449 | LYS62 | 0.275 | 0.0921 |
| THR2349 | 0.277 | 0.0213 | ASN70 | 0.314 | 0.0528 |
| ARG2351 | 0.279 | 0.0313 | VAL5 | 0.363 | 0.0749 |
| CYS2437 | 0.285 | 0.076 | SER74 | 0.38 | 0.0333 |
| SER2365 | 0.363 | 0.0803 |  |  |  |
| PRO2369 | 0.372 | 0.051 | Subunit C |  |  |
| GLY2311 | 0.379 | 0.1083 | ASP75 | 0.194 | 0.0388 |
| LEU2342 | 0.385 | 0.0471 | TYR102 | 0.232 | 0.0492 |
|  |  |  | ASN100 | 0.281 | 0.071 |
|  |  |  | GLU95 | 0.32 | 0.1518 |
|  |  |  | ARG72 | 0.361 | 0.13 |
| Subunit 3 |  |  | Subunit A |  |  |
|  |  |  |  |  |  |
| THR2349 | 0.181 | 0.0119 | ASP75 | 0.182 | 0.0121 |
| ARG2352 | 0.186 | 0.0193 | ARG72 | 0.205 | 0.0436 |
| GLN2356 | 0.205 | 0.0375 | LEU76 | 0.205 | 0.0188 |
| GLN2353 | 0.219 | 0.0331 | ASN34 | 0.207 | 0.0517 |
| GLN2435 | 0.228 | 0.0694 | THR2 | 0.215 | 0.0248 |
| LEU2342 | 0.229 | 0.0198 | ALA1 | 0.223 | 0.0733 |
| PRO2369 | 0.233 | 0.0211 | PRO73 | 0.226 | 0.0167 |
| VAL2367 | 0.236 | 0.0421 | GLY71 | 0.234 | 0.0244 |
| HIS2370 | 0.24 | 0.0258 | GLN66 | 0.242 | 0.044 |
| ARG2402 | 0.241 | 0.067 | LEU31 | 0.251 | 0.0418 |
| GLU2343 | 0.26 | 0.1213 | GLU35 | 0.254 | 0.0672 |
| TYR2309 | 0.275 | 0.0754 | SER68 | 0.26 | 0.0432 |
| SER2348 | 0.277 | 0.0245 | THR36 | 0.264 | 0.0828 |
| SER2365 | 0.297 | 0.0836 | GLN3 | 0.27 | 0.0296 |
| GLU2395 | 0.301 | 0.1248 | GLN64 | 0.319 | 0.1154 |
| ALA2350 | 0.319 | 0.0608 | ASN70 | 0.33 | 0.0992 |
| ILE2368 | 0.346 | 0.0471 | LYS62 | 0.378 | 0.1394 |
| GLN2364 | 0.35 | 0.081 | THR27 | 0.382 | 0.0995 |
| ASP2363 | 0.358 | 0.1152 | VAL5 | 0.396 | 0.1068 |
| ASN2310 | 0.369 | 0.119 |  |  |  |
| ASN2347 | 0.398 | 0.0599 | Subunit C |  |  |
|  |  |  | ASP75 | 0.188 | 0.0257 |
|  |  |  | ASN100 | 0.245 | 0.049 |
|  |  |  | TYR102 | 0.25 | 0.0432 |
|  |  |  | GLY97 | 0.361 | 0.1115 |

*Piezo numbering is given in accordance with uniprot E2JF22.

**Naming of subunits and numbering of LecB matches with those in PDB 4UT5.
